# Quantitative optical microspectroscopy, electron microscopy, and modelling of individual silver nanocubes reveal surface compositional changes at the nanoscale†

**DOI:** 10.1039/d0na00059k

**Published:** 2020-04-22

**Authors:** Yisu Wang, Attilio Zilli, Zoltan Sztranyovszky, Wolfgang Langbein, Paola Borri

**Affiliations:** School of Biosciences, Cardiff University Museum Avenue Cardiff CF10 3AX UK; School of Physics and Astronomy, Cardiff University The Parade Cardiff CF24 3AA UK langbeinww@cardiff.ac.uk

## Abstract

The optical response of metal nanoparticles is governed by plasmonic resonances, which depend often intricately on the geometry and composition of the particle and its environment. In this work we describe a method and analysis pipeline unravelling these relations at the single nanoparticle level through a quantitative characterization of the optical and structural properties. It is based on correlating electron microscopy with microspectroscopy measurements of the same particle immersed in media of different refractive indices. The optical measurements quantify the magnitude of both the scattering and the absorption cross sections, while the geometry measured in electron microscopy is used for numerical simulations of the cross section spectra accounting for the experimental conditions. We showcase the method on silver nanocubes of nominal 75 nm edge size. The large amount of information afforded by the quantitative cross section spectra and measuring the same particle in two environments, allows us to identify a specific degradation of the cube surface. We find a layer of tarnish, only a few nanometers thick, a fine surface compositional change of the studied system which would be hardly quantifiable otherwise.

## Abstract 2

A method and analysis pipeline correlating electron microscopy with quantitative micro-spectroscopy of particles immersed in different media is presented. Comparing measured and simulated cross sections reveals a tarnish layer on silver nanocubes.

## Graphical abstract

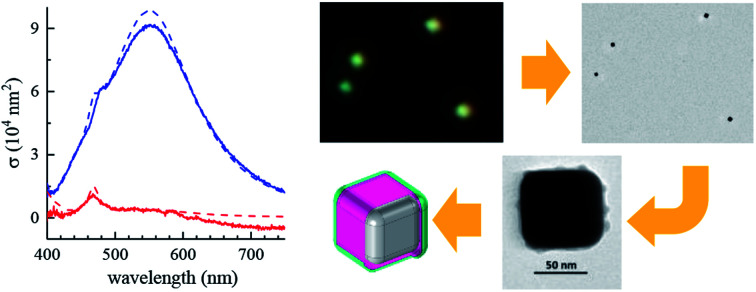

## 1 Introduction

The remarkable optical properties of subwavelength metal nanoparticles (henceforth particles) have fascinated scientists for centuries, the first reports on metal colloids dating back to M. Faraday.^1^ These properties are understood in terms of localized surface plasmon resonances (LSPRs), which rule their optical response.^2^ The interest in this research topic is driven by the sensitive dependence of the LSPRs on the parameters of the system such as particle composition, shape and size, as well as the nature of its environment.^3^ Such complexity on the one hand poses significant experimental and technological challenges, and on the other hand offers ample opportunities for tailoring the optical properties to the needs of different applications.^4^ This fostered the fabrication of metal particles with diverse morphologies – such as spheres, rods, tetrahedra, and platelets – relying on the versatility and cost-effectiveness of colloid chemistry methods.^5^ Among those, cube colloids can be produced with exceptional crystalline quality and low dispersity; moreover, the sharp corners and flat faces of cubes are interesting for several applications, as discussed further below.

Silver excels among plasmonic materials thanks to its low ohmic losses, which yield sharp LSPRs over the wavelength (*λ*) range 400 to 1200 nm.^3,5^ The reactivity of silver surfaces underpins applications in the rapidly growing field of nanocatalysis.^6,7^ In particular, it has been observed that irradiation at the LSPR frequency enhances the catalytic activity of silver cubes.^8,9^ The LSPR dependence on the local environment is exploited in numerous dielectric sensing schemes,^3,6^ and the sharp resonances of silver particles can offer a high sensitivity.^10,11^ The strong and highly localized field enhancement at the LSPR makes metal nano-particles suitable candidates for surface-enhanced Raman scattering platforms.^12^ For example, Matteini *et al.* detected the Raman emission of proteins in the field hot-spots at the corners of colloidal silver cubes.^13^ The atomically flat faces of cubes allow realizing plasmonic cavities of nanometric thickness in dimers^14,15^ or between the cube and a metal substrate.^16^ The strong coupling of molecules or quantum dots to the cavity modes drastically enhances the spontaneous emission rate, paving the way for single molecule detection and bright single photon sources.^17,18^

The design of particle systems^19^ for applications such as the ones just outlined must be grounded in a thorough understanding and a quantitative modelling of the often complex relation between the optical properties and morphology. These studies are best performed at the single particle level, to avoid effects of sample dispersity. The optical properties of individual cubes have been addressed through far-field optical microscopy,^11,20–22^ while electron energy loss spectroscopy provided a nanometric mapping of the plasmonic modes.^23,24^ In single particle studies, the measured optical spectra are usually compared to theoretical calculations or numerical simulations.^25^ By adjusting the parameters of the model to match the measurements, one can gain relevant information on the system; in practice, however, the parameters are often too many for this procedure to provide univocal results. It is useful, in the first place, to constrain the parameter space through a precise geometrical characterization of each investigated particle *via* transmission electron microscopy (TEM). However, some structural details and material properties, such as the contamination or chemical alteration of the particle surface, which is difficult to avoid,^26^ are hardly assessed with TEM, and yet can have a sizeable impact on the optical properties as well as on the catalytic activity. In this article, we show how the comparison between the experiment and theory can be made more stringent by gathering and taking into account the cross section magnitude additionally to the previously considered LSPR position and linewidth, and correlating measurements of the same particle in different environments.

The cross sections for optical scattering (*σ*_sca_) and absorption (*σ*_abs_) quantify the strength of the interaction of a particle with light.^2^ Presently, only a few experimental techniques are capable of measuring, with significant limitations, the cross section magnitude at the single particle level.^4,27^ The main tool available is spatial modulation spectroscopy (SMS),^28^ which addresses the extinction cross section *σ*_ext_ = *σ*_sca_ + *σ*_abs_; this implies it is only accurate for absorption-dominated particles, unless some correction for the fraction of the total scattering collected is applied as in ref. 15. A quantitation of *σ*_abs_ can be obtained with photothermal methods,^29^ but requires a precise modeling of the thermal properties of the particle environment. As for *σ*_sca_, just a handful of quantitative measurements can be found in the literature, which refer to large (≃200 nm), scattering-dominated particles.^15,30^ Of note, interferometric scattering microscopy (iSCAT)^31^ is capable of high-sensitivity scattering detection, and is used for negligible *σ*_abs_ to quantitatively determine the mass of small dielectric particles down to the single protein limit.^32^

We recently demonstrated^33^ a measurement and data analysis method for accurate quantification of both *σ*_sca_ and *σ*_abs_ at the single particle level. In comparison to the other single-particle techniques listed above, the method requires only relatively affordable equipment: an optical microscope equipped with a white light source and coupled to a spectrometer. Here, we present an enhanced version of the quantitative analysis, making full use of numerical modelling, which expands the scope of the approach to particles of arbitrary composition, shape, and size above the electrostatic limit. It furthermore models the three-layer structure of the TEM-compatible sample mount needed for the correlative workflow, and the realistic excitation and collection angular ranges. We study silver cubes, which, as mentioned above, are interesting particles in their own right, as well as providing an example of the degree of knowledge to be gained through the presented correlative and quantitative approach.

## 2 Experimental

**Fig. 1:**
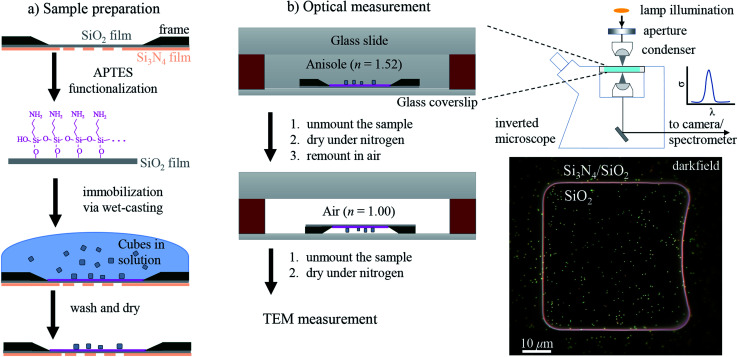
Schematic workflow of the experiment. (a) Sample preparation steps: functionalization (purple layer) of the TEM grid followed by wet-casting of the cube colloid. The grid is composed of a 40 nm-thick SiO_2_ film supported by a 200 nm-thick Si_3_N_4_ film with 50 × 50 μm^2^ square windows. (b) Sample mounting for correlative optical microspectroscopy, first in anisole and then in an air environment. On the right, a sketch of the optical setup and a dark-field image of the sample showing a SiO_2_ window with deposited cubes is shown.

**Fig. 2:**
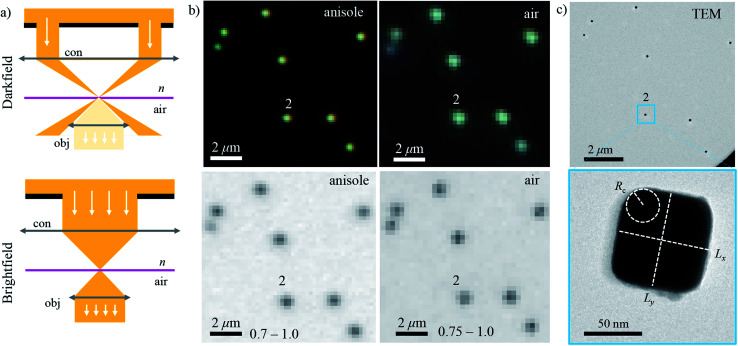
Correlative optical-electron imaging of silver cubes. (a) Sketch of the DF (above) and bright-field (BF) (below) illumination schemes. (b) DF (top row) and BF (bottom row) micrographs of the sample immersed in anisole (left column) or in air (right column). The DF images were acquired with a commercial colour camera (Canon, EOS 40D), whereas the BF images with a scientific CMOS camera (PCO Edge 5.5). The BF grey scale range (black to white) is given next to the length scale bar. (c) TEM micrograph (above) of the same region as shown in panel (b), and (below) a close-up on an individual cube (#2). A magnification of 2.5k and 80k was used, respectively.

The workflow of the experiment is presented in Fig. 1. Silver cubes of 75 nm nominal edge size were deposited on a silica (SiO_2_) film suitable for both optical and electron microscopy. As detailed in Section 2.1, the silica surface was functionalized to bind the silver cubes. This allowed changing the immersion medium without displacing the particles and thereby correlating optical measurements in different environments with electron microscopy in vacuum. As depicted in Fig. 1b, optical spectra were first acquired in anisole (methoxybenzene, a volatile liquid of refractive index *n* = 1.52, matched to glass) and then in air (*n* = 1.00), as detailed in Section 2.2. TEM on the measured particles, as detailed in Section 2.3, was the final experimental step, to prevent potential damage by the electron beam (see Fig. S1 of the ESI†) from affecting the optical measurements. Fig. 2 shows that the particle positions correlate well between the three measurements, confirming they are stably immobilized on the substrate.

The experimental set-up and settings used for microspectroscopy measurements are described in Section 2.2. The magnitude of *σ*_sca_(*λ*) and *σ*_abs_(*λ*) is measured^33^ correlating a scattering and a transmission measurement of the same particle. To do so, two illumination modalities are alternated: dark-field (DF) for scattering and bright-field (BF) for transmission, which are sketched in Fig. 2a. In DF, the illuminated numerical aperture (NA) range of the condenser lens (or illumination cone) is above the NA of the objective, so that no direct light is collected, rendering the background dark. Particles and debris scatter some light into the detection cone given by the NA of the objective, and thus appear as bright spots. By contrast, in BF, the illumination and detection NA ranges are set to be equal, so that the background is bright, with particles appearing as dark spots because they remove some of the transmitted light from the detection cone through absorption and scattering. Correlated DF–BF micrographs which exemplify the discussed features are shown in Fig. 2b.

In order to extract the magnitude of *σ*_sca_ and *σ*_abs_ from the correlated images, some previous knowledge of the optical properties of the studied system is required. Specifically, one needs to know (for details see Section S.VI in the ESI†): (i) the angular distribution 
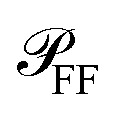
 of the scattered power in the far field (FF), which is required to calculate the fraction *η* of light scattered into the detection cone of the objective; (ii) the dependence of 
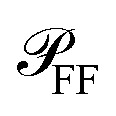
 on the illumination modality, from which the BF-to-DF ratio of total scattered power, called *ζ*, is determined. We emphasize that 
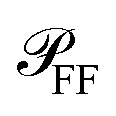
 as well as *σ* depend not only on the particle and its environment, but also on the excitation employed, particularly its polarisation and direction of incidence, which determine the orientation of the exciting electric field of the particle. For instance, high-NA illumination provides a significant field component along the axis of the microscope. For this reason in the following sections we will indicate with the superscript *l* ∈ {BF,DF} the excitation condition for the quantities depending on it, such as *σ*^BF^_abs_ and *σ*^DF^_sca_.

In ref. 33, the two scattering parameters *η*^l^ and *ζ* required for quantitation were calculated through an analytical model using approximations regarding the geometry of the particle and its environment, mainly assuming a dipolar response and neglecting multiple scattering events. Generally speaking, these approximations are accurate for small particles (≪*λ*) of simple shape. In the present work, *η*^l^ and *ζ* are calculated fully numerically instead, dropping the approximations of the analytical approach, and thereby improve the accuracy and the generality of the quantitative method. At the same time, the numerical simulations provide the optical cross section spectra for a given particle description, which can be compared with the experiments.

The numerical model is described in detail in Section 2.4. To reproduce the experiment accurately, we modelled each cube using its geometry determined by TEM. The large flat surfaces of the cubes encourage them attaching with one surface parallel to the substrate, so that in plane-view TEM two dimensions of the cube, called *x* and *y*, can be assessed. As depicted in Fig. 2c, the face-to-face distances *L*_*x*_ and *L*_*y*_, and the radius of edge curvature *R*_c_ averaged over the four corners were extracted from the TEM micrographs. The cube size in the dimension orthogonal to the substrate could not be assessed with TEM and the arithmetic mean *L*_*z*_ = (*L*_*x*_ + *L*_*y*_)/2 was taken.

Most numerical simulations in the field of nanophotonics employ plane wave (PW) excitation, as this is the simplest to implement. However, this simplification fails to account for the varying propagation directions and polarisations contained in a high NA illumination cone. Consequently, cross section spectra computed under PW excitation and microscope illumination can differ significantly, as will be shown below. An analytical framework to reproduce the incoherent, high-NA illumination of microscopy experiments by interpolating multiple PW simulation results within the illumination cone was developed, as given in Section S.V of the ESI.† The same approach is applied to compute 
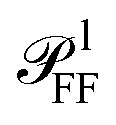
 and hence *η*^l^ and *ζ*; see Section S.VI A of the ESI.† The specific directional weighting used for averaging derives from a mathematical description of the incoherent microscope illumination reported in Section S.IV of the ESI.† This description represents the condenser as an aplanatic optical system and includes the intensity profile in the back focal plane (BFP) of the lens, which has been characterized experimentally; see Fig. S7 of the ESI.†

It is appropriate to highlight some conceptual aspects of the meaning of the cross sections reported in this article. In general, cross sections are defined as the power *P* removed from the exciting electromagnetic mode(s), referenced to an incident intensity *I*_0_, so that *σ* = *P*/*I*_0_. When the excitation is a PW, the identification of *I*_0_ is straightforward. For an incoherent microscope illumination, on the other hand, the excitation contains many mutually incoherent modes. In this scenario, we define *I*_0_ as the intensity *I*_FFP_ impinging from the top medium on the front focal plane (FFP) of the condenser, where the sample is placed for imaging. This is a natural operative definition because a signal proportional to *I*^BF^_FFP_ (*via* the transmission *τ*^BF^ of the substrate) is measured in BF in absence of particles. Furthermore, this definition also reduces to the PW definition in the limit of small illumination NA. Notably, due to the projection onto the FFP, high angles of incidence correspond to a lower *I*_FFP_ and therefore result in a larger *σ*. The resulting dependence of *σ* on the angular range of illumination, dubbed “long shadow effect”, has been discussed and verified experimentally in ref. 34.

We emphasize that *I*^DF^_FFP_ cannot be measured directly, as the illumination is not detected in DF. However, the ratio *ξ* ≡ *I*^BF^_FFP_/*I*^DF^_FFP_ depends only on the NA ranges of BF and DF. The illumination parameters *τ*^BF^ and *ξ* do not depend on the particles investigated and can be calculated analytically as shown in Section S.VI B of the ESI.†

### 2.1 Sample preparation

The sample preparation steps for correlative studies are depicted in Fig. 1. The particles were deposited on a silicon TEM grid (Ted Pella, 21530-10) composed of a 40 nm-thick SiO_2_ (silica) film supported by a 200 nm-thick Si_3_N_4_ film with 50 × 50 μm^2^ square windows. The grid was cleaned with deionized water followed by acetone, then anisole, and then dried in air. After cleaning, the grid was etched at 55 °C for 1 h in a 10 mL solution of 500 μL H_2_SO_4_ (98%) diluted in 9.5 mL of 30% H_2_O_2_, and then it was thoroughly washed with deionized water. The silica surface was functionalized adapting the protocol of ref. 35: the etched grid was incubated in a solution of 1% (v/v) 3-aminopropyl triethoxysilane (APTES) (Sigma Aldrich) in ethanol for 1 h, then washed three times in ethanol followed by three times in water, and finally dried in air.

Silver cubes of 75 nm nominal edge size were purchased from nanoComposix (NanoXact, SCPH75-1M). The colloid is stabilized by polyvinylpyrrolidone (PVP), a polymer which reduces aggregation in solution and suppresses the reactivity of the silver surface.^5^ The cubes were deposited on the TEM grid with a wet-casting protocol yielding a strong attachment on the functionalized surface with a homogeneous coverage. The stock colloid was diluted to 0.1 optical density (at *λ* = 515 nm with 1 cm path length) in order to achieve a surface density of about 0.1 particles per μm^2^, which is low enough to optically resolve individual particles, while at the same time high enough to find a few particles within the 5 × 5 μm^2^ field of view of the TEM (at 2500 magnification as shown in Fig. 2c) and recognize a pattern for correlation. Further details on the wet-casting protocol and the concentration optimization are given in Section S.I of the ESI.† 9 μL of the diluted colloid were dropped onto the TEM grid and incubated at room temperature for 15 min; drops of deionized water were added in the meantime to prevent a complete evaporation of the sample solution. Afterwards the sample grid was washed three times in water and dried under a nitrogen flow.

### 2.2 Optical measurements

To mount the sample for optical measurements, a 0.5 mm thick press-to-seal silicone spacer (Grace Bio-Labs, 664507) was used to create a chamber between a coverslip and a glass slide where the sample grid could be accommodated within the immersion medium as depicted in Fig. 1b. Microspectroscopy was performed using a commercial inverted microscope (Nikon, Eclipse Ti-U) featuring some customization. A tungsten-halogen lamp (Nikon, 17005M28) provides unpolarised and incoherent illumination, which is focused onto the sample by using a 1.34 NA oil-immersion condenser lens (Nikon, T-C-HNAO). The NA range of the illumination is defined by suitable light stops placed in the BFP of the condenser. The stops are mounted on a slider which permits to rapidly switch between the DF and BF illumination modalities depicted in Fig. 2a with no need to move the condenser, as is required for precise correlation of scattering and transmission images. A diffuser (Thorlabs, ED1-C20) is inserted in the illumination path before the field iris to provide a nearly homogeneous illumination over the BFP of the condenser. The light intensity profile in the BFP was characterised; see Fig. S7 in the ESI,† because this information is required for quantitative measurements. Light is collected using dry microscope objectives (Nikon, CFI plan apochromat λ series) of 0.95 NA for measurements in anisole and 0.75 NA for measurements in air. In DF, illumination cones ranging from 1.10 to 1.34 NA in anisole and from 0.80 to 0.90 NA in air were used. In BF, the illumination cones matched the detection NA range given by the objective.

The microscope output is optically coupled to an imaging spectrometer (Horiba Jobin-Yvon, iHR550) equipped with a ruled plane diffraction grating (Horiba, 51048) of 78 mm side length and 100 lines per mm. Spectra are recorded by using a Peltier-cooled back-illuminated charge-coupled device (CCD) sensor (Andor, Newton DU-971N). DF spectra were acquired with an exposure time of 3 s. Note that the DF sensitivity is limited by the diffuse scattering background produced by small debris and out-of-plane scatterers, rather than by the detector noise. We acquired a local DF background for each particle, which was subtracted from the scattering signal. This background creates a shot noise limit of about 10 nm^2^ for *σ*_sca_ per spectral channel of about 0.5 nm width, while at the peak of the scattering intensity the shot noise is increased to about 50 nm^2^. BF instead provides a much lower contrast, and its sensitivity is limited by the shot noise of the transmitted light background. We therefore accumulated 50 transmission spectra with an individual exposure time of 0.3 s, to provide a shot noise in *σ*_ext_ of about 200 nm^2^ root mean square per spectral channel. These numbers vary somewhat with wavelength due to the spectral dependence of the illumination intensity. They are quoted for the measurements in anisole and are about twice larger in air. Note that the shot noise is below 1% of the typically measured cross-sections, so that systematic errors are expected to dominate the measurement accuracy.

The experimental set-up and measurement procedure are presented in greater detail in the ESI of ref. 33, specifically in Sections S.II and S.VI.

### 2.3 Electron microscopy

Images were acquired with TEM (JEOL, JEM-2100) at a magnification of 80k, a beam current of 100 μA and an acceleration voltage of 200 kV, using a high-resolution CCD camera (Gatan). Analyzing micrographs of the same cubes taken with different magnifications (60k, 80k, and 100k), a RMS variation of 3% of the TEM scaling calibration was found. This results in a ±1 nm estimated accuracy of the measurement of *L*_*x*_ and *L*_*y*_. It is reasonable to assign the same uncertainty to *R*_c_, considering some degree of arbitrariness in drawing the circle interpolating the corner as shown in Fig. 2c.

### 2.4 Numerical modeling

The simulated geometry is presented in detail in Section S.III of the ESI.† Briefly, the silver cube lies flat on the TEM grid window, which is modeled as a *h* = 40 nm thick slab of silica (*n* = 1.46) perpendicular to the optical axis of the microscope. The immersion medium on both sides of the slab is either anisole (*n* = 1.52) or air (*n* = 1.00). Silver is described by the wavelength-dependent permittivity reported by Yang *et al.*^36^ (single crystal data set), which is more accurate that the commonly used one by Johnson and Christy,^37^ specifically regarding the imaginary part. The PVP coating (*n* = 1.53) is modelled as a shell of 2 nm homogeneous thickness.

Apart from the three-layered environment and the particle geometry, the numerical model is described in Section S.II of the ESI of ref. 33. Briefly, we simulate scattering and absorption using the frequency-domain formulation of the electromagnetic problem and the finite element method as implemented in the commercial solver COMSOL Multiphysics®. The electromagnetic excitation is a PW propagating in a given direction within the illumination cone. It is created using its analytical expression in each medium, derived in Section S.II of the ESI.† Different from ref. 33, we also compute numerically the scattering parameters needed for quantitative measurements. To do so with the same simulations giving *σ*_sca_ and *σ*_abs_, we also computed the angular distribution of scattered power 
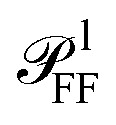
*via* the near-to-far field transform (Stratton-Chu formula) provided by the electromagnetic module of COMSOL.

We reproduce the high NA, incoherent illumination by averaging multiple PW simulations with different directions of incidence sampling the illumination cone. Exploiting the fourfold symmetry of the system, we sampled just one quadrant of the NA plane with 35 simulations; see details on p. S19 of the ESI.† The unpolarised illumination is reproduced by averaging the results of two PW simulations having the same direction of incidence and orthogonal (p and s) polarisation. To obtain an adequate spectral resolution, we computed 77 wavelength values, so that 35 × 2 × 77 = 5390 model runs were required to obtain one simulation under microscope illumination. The model is solved in about 15 s on a modern workstation (Intel Core i7-5930K CPU), so that about 22 h of computation are needed. This is then repeated for 11 cubes, each in 2 environments. Such a large number of simulations mandates extensive automation, which we realized using the LiveLink™ to MATLAB of COMSOL to run the multiparametric sweep, post-process the simulation results and store them on-the-fly.

## 3 Results and discussion

### 3.1 Optical microspectroscopy

**Fig. 3:**
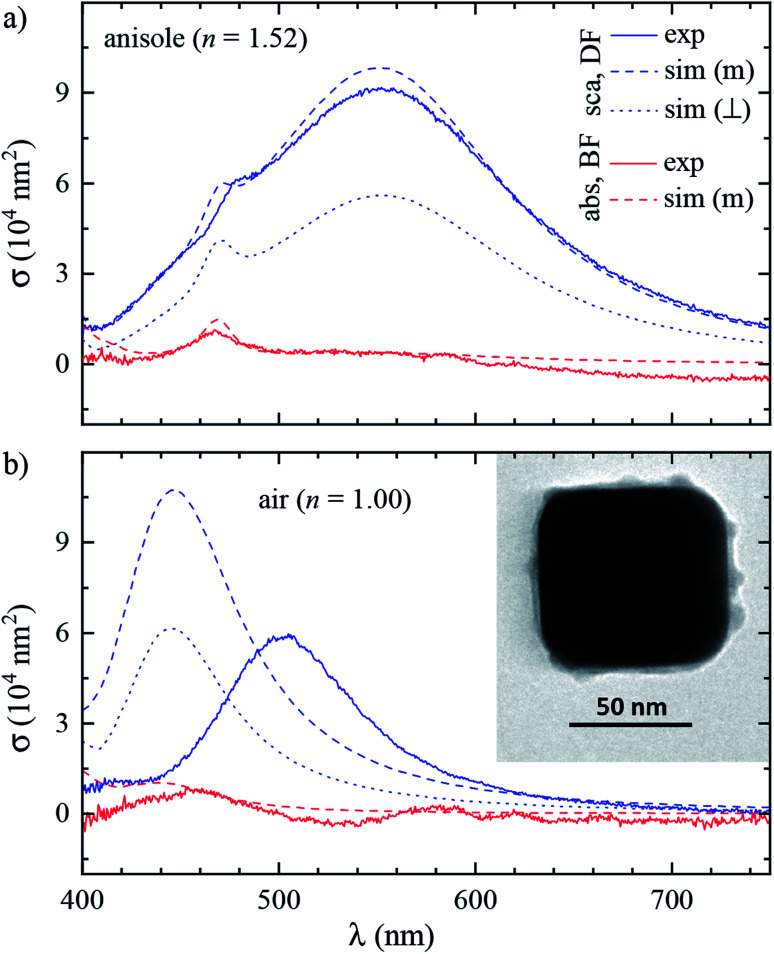
Spectroscopy of a single cube (#4). Scattering (blue) and absorption (red) cross section spectra in (a) anisole and (b) air environment. Solid lines are experiments. Simulations are performed under either (dash) microscope illumination (m) or (dot) PW illumination at normal incidence (⊥).

Fig. 3 shows the measured and simulated cross section spectra of a single cube (#4). Its geometrical parameters, determined from TEM (inset of panel (b)), and used in the numerical simulations, are *L*_*x*_ = 75.4 nm, *L*_*y*_ = 71.6 nm, and *R*_c_ = 14.1 nm. Let us discuss the main spectral features starting with scattering in anisole (panel (a)).

*σ* ^DF^_sca_(*λ*) is dominated by a broad dipolar (D) resonance at *λ*_D_ ≈ 552 nm, and a quadrupolar (Q) resonance appears as a shoulder at *λ*_Q_ ≈ 470 nm. The Q resonance is sharper due to a lower radiative coupling,^11^ resulting in a lower damping by radiation to the FF. *σ*^DF^_sca_(*λ*) is simulated under two different illuminations: a plane wave propagating perpendicular (⊥) to the silica film, *i.e.* along *z*, and the high NA incoherent microscope (m) illumination used in the experiment, modelled through the angular averaging procedure described in Section 2.4. The m simulation is in good quantitative agreement with the experiment, whereas for ⊥ illumination the D peak is almost halved and the Q shoulder is more pronounced than in the experiment. These sizable differences highlight the importance of accurately modelling the experimental illumination for quantitative analysis. Let us now turn our attention to *σ*^DF^_sca_(*λ*) in air, shown in Fig. 3b. The lower refractive index brings about a blue shift of *λ*_D_ to 502 nm. Yet, the simulated D peak is blue-shifted by a further 60 nm with respect to the experiment; we will come back to this discrepancy later.

As for the absorption in anisole in Fig. 3a, interestingly *σ*^BF^_abs_(*λ*) does not feature the D resonance, with good agreement between the experiment and simulation. This results from the large radiative coupling (scattering) of the D resonance, which provides a decay pathway to the electronic excitation much more efficient than internal losses (absorption).^38^ Furthermore, *λ*_D_ is well above the plasma wavelength of silver of about 330 nm, resulting in a small imaginary part of the permittivity relative to its real part^36^ of about 4% at *λ*_D_, reducing the material absorption.^39^ Concerning the systematics in *σ*^BF^_abs_(*λ*), particularly apparent in the negative cross section values at long wavelengths, we note that in the experiment *σ*^BF^_abs_ is determined by subtracting the scattering contribution *σ*^BF^_sca_ = (*ζ*/*ξ*)*σ*^DF^_sca_ from the BF extinction cross section *σ*^BF^_ext_; see eqn (7a) of ref. 33. The error of *σ*^BF^_abs_ is therefore obtained by propagating the errors of *σ*^DF^_sca_, *σ*^BF^_ext_, and of the parameters *ζ* and *ξ* required to retrieve *σ*^BF^_sca_ from *σ*^DF^_sca_. If *σ*^BF^_abs_ is much smaller than *σ*^BF^_ext_, the resulting error can be larger than the value of *σ*^BF^_abs_. Considering these errors, which we estimate to be of the order of 10% for each quantity, the simulation and experiment are consistent, showing that the D resonance cross section is dominated by scattering. Notably, the Q resonance shows a significant absorption, of similar magnitude as its contribution to the scattering, in both simulation and experiment. This can be understood with the same arguments as for the D resonance. The Q resonance has a lower radiative coupling than the D resonance, and the ratio between the imaginary and real part of the permittivity at *λ*_Q_ is higher, about 5%.

**Fig. 4:**
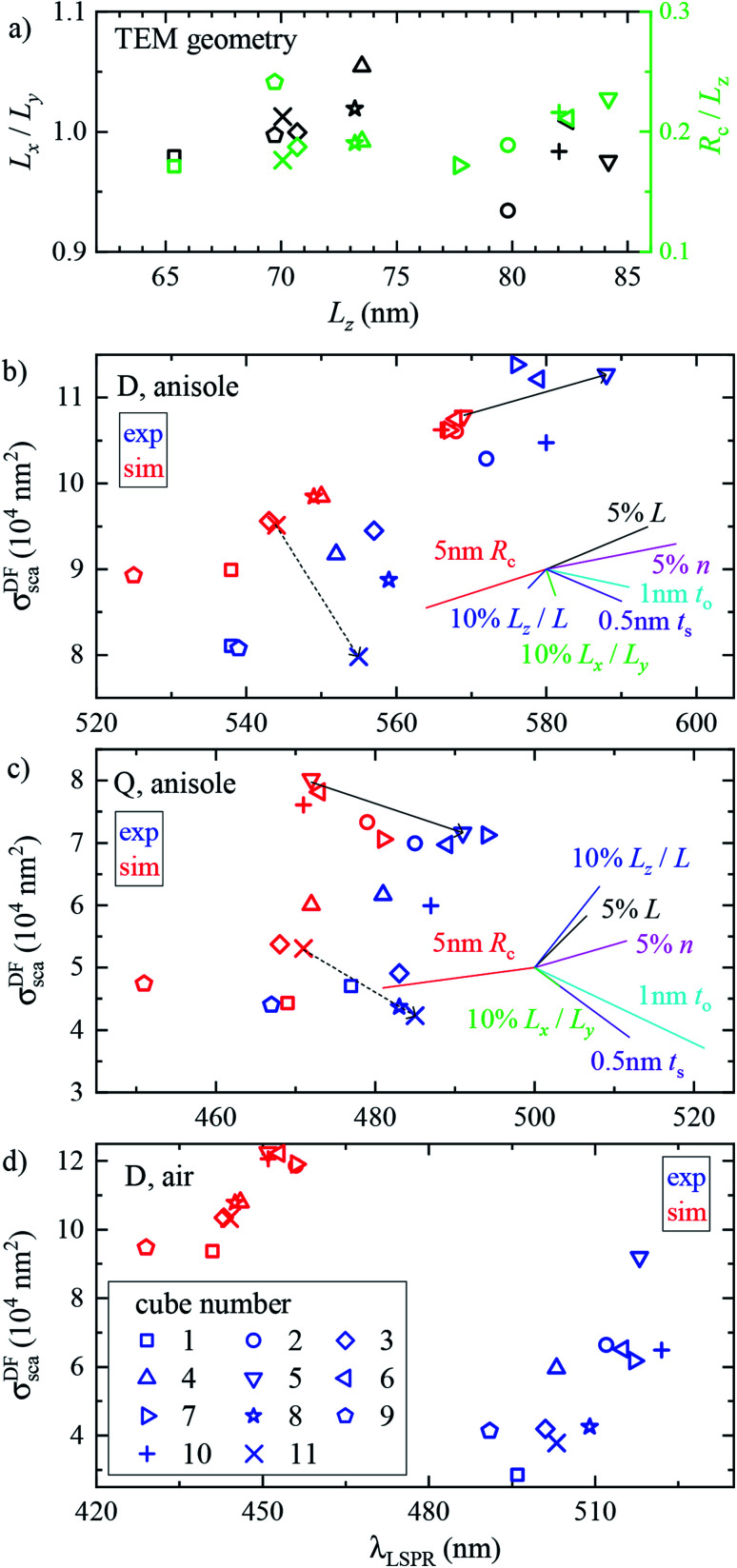
Geometrical and optical properties across the investigated set of cubes. Individual cubes are identified by different symbols according to the legend in panel (d). (a) TEM characterization: in-plane asymmetry *L*_*x*_/*L*_*y*_ (black symbols) and edge rounding *R*_c_/*L*_*z*_ (green symbols), *versus* mean size *L*_*z*_. (b)–(d) Experimental (blue) and simulated (red) magnitude *σ*^DF^_sca_ (*λ*_LSPR_) *versus* resonance position *λ*_LSPR_ of (b) the D peak in anisole, (c) the Q peak in anisole, and (d) the D peak in air. In (b) and (c) the arrows indicate the difference between the experiment and simulation for the two cubes. The coloured lines show the relative changes resulting from the increase of various simulation parameters from a reference, as indicated by the labels. The origin of the lines is chosen so as to avoid overlap with the data.

We performed analogous correlated studies on 11 cubes in total. The TEM characterization is summarized in Fig. 4a, while numerical parameter values are given in Table S1 of the ESI.† The mean size *L*_*z*_ = (*L*_*x*_ + *L*_*y*_)/2 shows a ±10 nm dispersity with respect to the nominal value of 75 nm, and the in-plane asymmetry *L*_*x*_/*L*_*y*_ − 1 is below 5% for all cubes. The edge rounding *R*_c_/*L*_*z*_ is (20 ± 5)%, corresponding to *R*_c_ values in the range 11 to 19 nm. No clear correlation between size, asymmetry, and edge rounding is observed. The measured and simulated spectra of all cubes are shown in Fig. S8–S10 of the ESI.† Notwithstanding some variability between cubes, the above discussion of the cross section spectra applies qualitatively to all. Fig. 4b–d summarize the spectral information, by reporting the position and magnitude of the resonances. The simulated D peaks in anisole (Fig. 4b) are in average about 10% larger and 15 nm blue-shifted with respect to the experiment. Similar differences are found for the Q resonance in anisole (Fig. 4b). Stronger deviations are observed for the D resonance in air (Fig. 4d): the simulated peaks are about twice as large as the measured ones, and blue-shifted by approximately 60 nm. We estimate that systematics can affect the measured magnitude of *σ*_sca_ up to 10% in anisole and 20% in air. These mostly derive from the uncertainty in the illumination NA range used in the DF measurements: in air a narrower range was used (see Section 2.2), resulting in a larger uncertainty. For further details, we refer the reader to the discussion of error sources in ref. 33. The measured peak position is accurate within a few nm. We therefore ascribe the observed deviations mostly to a difference between the simulated and measured particle structure, which will be discussed in the following sections.

### 3.2 Exploring the simulation parameters: geometry and permittivity

**Fig. 5:**
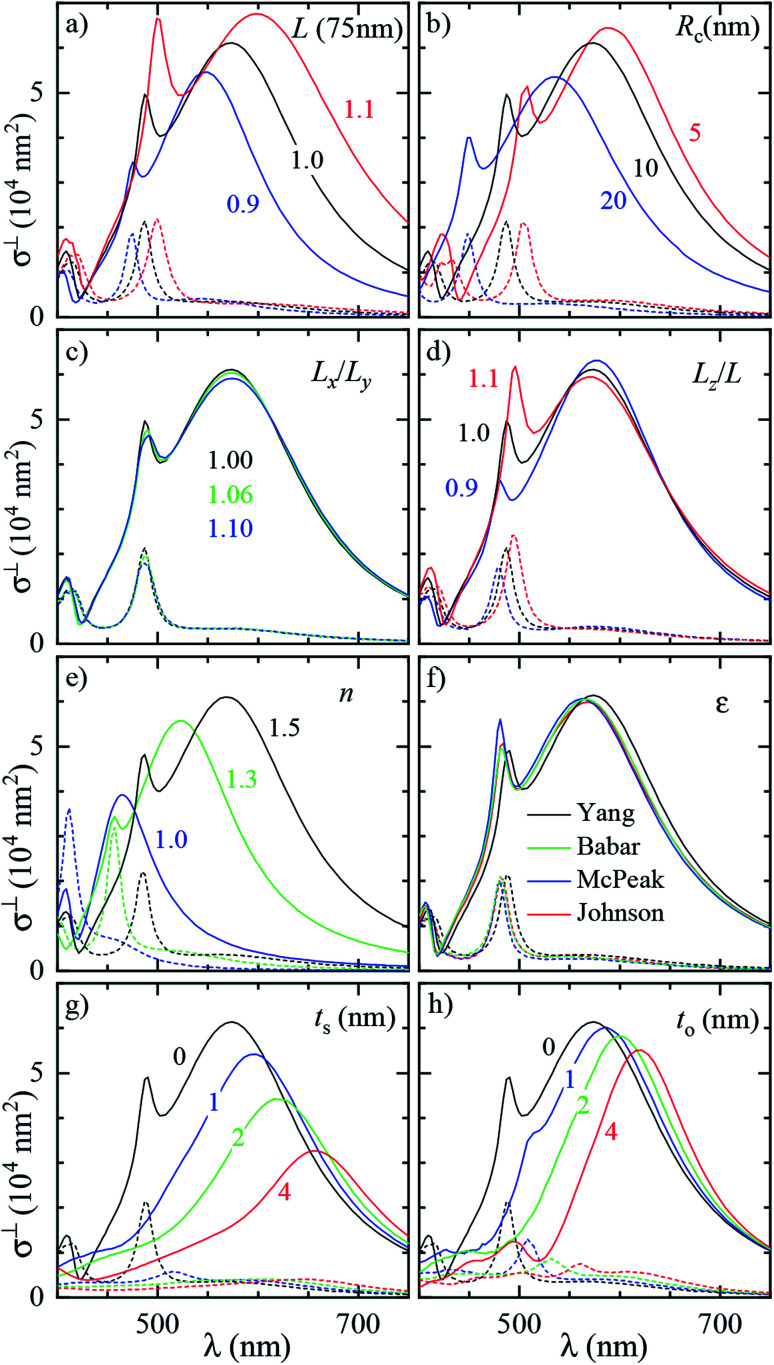
Dependence of the simulated scattering *σ*^DF^_sca_(*λ*) (solid lines) and absorption *σ*^BF^_abs_(*λ*) (dashed lines) cross section spectrum on various parameters of the system. Simulations were performed under ⊥ illumination starting from a reference cube (*L* = 75 nm, *R*_c_ = 10 nm) immersed in anisole (*n* = 1.52). The following parameters are varied: (a) size of the cube *L* (isotropic scaling so that *R*_c_ varies); (b) radius of edge curvature *R*_c_; (c) in-plane aspect ratio *L*_*x*_/*L*_*y*_ (keeping *L*_*x*_*L*_*y*_ = *L*^2^); (d) height aspect ratio *L*_*z*_/*L* (changing *L*_*z*_); (e) refractive index *n* of the immersion medium; (f) Ag permittivity according to different reports^36,37,41,42^ as indicated by the legend; (g) thickness *t*_s_ of the Ag_2_S layer; (h) thickness *t*_o_ of the Ag_2_O layer.

To assess the significance of the differences between measured and simulated properties observed in Fig. 3 and 4, let us first see how much they are affected by the uncertainties of the characterization of the system. In Fig. 5 we systematically study the effect of varying the parameters of the modelled system. A cube of *L* = 75 nm and *R*_c_ = 10 nm immersed in a homogeneous medium of refractive index *n* = 1.52 (such as anisole) is chosen as the reference description. To reduce the computational workload, these simulations were performed under normal incidence (⊥) PW excitation, with the view that the dependencies under microscope (m) illumination are similar. The variation of the position and magnitude of the D and Q resonances in the scattering cross section is reported in Table 1.

**Table 1:** Effect of varying the parameters of the modeled system on the D and Q resonance in the scattering cross section

Fig. 5 panel	Parameter variation	Δ*λ*_D_ (nm)	Δ*σ*^⊥^_sca_(*λ*_D_) (%)	Δ*λ*_Q_ (nm)	Δ*σ*^⊥^_sca_(*λ*_Q_) (%)
a	*L* × 1.01	+2.7	+1.1	+1.3	+3.3
b	*R*_c_ + 1 nm	−3.2	−1.0	−3.8	−1.3
c	*L*_*x*_/*L*_*y*_ × 1.01	+0.12	−0.34	+0.31	−0.59
d	*L*_*z*_/*L* × 1.01	−0.24	−0.25	+0.81	+2.6
e	*n* × 1.01	+3.5	+0.65	+2.3	+1.7
g	*t*_s_ + 1 nm	+20	−12	+24	−46
h	*t*_o_ + 1 nm	+11	−2.4	+21	−26

In Fig. 5a the cube is scaled isotropically (that is, *R*_c_ varies too). By increasing *L* by 10%, the D peak red-shifts by 27 nm, *i.e.* with a slope of 2.7 nm/%. Such a red shift is due to retardance effects: indeed, the size parameter 2π*nL*/*λ*_D_ ≃ 1.2 is not small. At the same time, the D peak increases in amplitude with a slope of 1.1%/% (percent increase of *σ*_sca_ per percent increase of *L*). It is interesting to compare this value to the theoretical scaling: *σ*_sca_ ∝ *L*^6^ in the electrostatic limit (*L* ≪ *λ*) and *σ*_sca_ ∝ *L*^2^ in the geometric limit (*L* ≫ *λ*). The predicted slope is then 6.2%/% and 2.0%/% respectively, higher in both cases than the simulated value. We ascribe the reduced scaling to the increasing radiative damping, which broadens the resonance, thus reducing its magnitude.^40^ The Q peak red-shifts less than the D peak, but increases more; see the first row of Table 1. The changes in the peak position and amplitude upon increasing various system parameters are represented by coloured lines in Fig. 4b (D resonance) and Fig. 4c (Q resonance) to visually aid the comparison with the observed differences. In particular, the black line indicates the changes due to a 5% increase of *L*. The dependence on *L* appears too weak to account for the observed deviations from the experiment within the 2% uncertainty attributed to TEM measurements.

A few nm smaller edge rounding *R*_c_ entails instead a sizable red shift of both resonances (see Fig. 5b) which could improve the agreement for many cubes. Using slightly sharper edges in the model can be justified because TEM imaging is the last experimental step, and edges tend to round up over time as they have a large surface energy. Note, however, that sharpening the edges brings about some increase of *σ*_sca_ as well, whereas the simulated values in Fig. 4 are already larger than in the experiment.

The in-plane aspect ratio *L*_*x*_/*L*_*y*_ weakly affects the simulated spectra (see Fig. 5c), albeit in a direction which brings them closer to the experimental data. Specifically, an agreement would be reached for aspect ratios of 1.3 and above, but they are well beyond the uncertainty of the TEM measurements.

The height aspect ratio *L*_*z*_/*L* is not measured by TEM and thus its tuning can be justified to some extent, but it has a strong effect only on the Q peak; see Fig. 5d.

Increasing the refractive index *n* red-shifts both resonances; see Fig. 5e. However, the *n* of anisole is known with good precision (better than 1%) and is dispersed by just 2% over the investigated spectral range. Larger variations of *n* could only be justified as a modification of the local environment of the cube, a hypothesis which will be discussed separately in the following sections. Furthermore, increasing *n* to red-shift the simulated data points toward the experimental ones in Fig. 4 increases their magnitude, which is already larger than the experiment.

Modeling silver with different permittivity data sets from the literature^36,37,41,42^ brings about little change to the spectra; see Fig. 5f. Specifically, the D peak varies over a range of 12 nm in position and 2.6% in magnitude, while the Q peak varies over a range of 7 nm position and 14% in magnitude. Moreover, the resonance linewidth agrees well between the experiment (141 ± 25) nm and simulation (139 ± 28) nm; see Table S1 of the ESI.† The FWHM values are given as mean ± one standard deviation over the 11 investigated cubes and were extracted *via* a Lorentzian fit of the D peak alone. Such good agreement confirms that a bulk permittivity data set is adequate to describe particles larger than few tens of nm (≃electron mean free path), where surface damping mechanisms^40,43^ play a negligible role.

To summarize, *R*_c_ is the sole model parameter which is able to significantly improve the agreement with a realistic variation (a few nm sharpening of the edges). However, the experimental peak magnitude would still be too low, which could at this point only be ascribed to systematics affecting the cross section magnitude measurements. Summarizing, it appears that varying the system parameters within the uncertainties of the structural characterisation is not sufficient to accommodate the differences between the experiment and simulation observed in anisole. Nor, *a fortiori*, can one expect it to explain the much larger discrepancies observed in air. This indicates that the model does not capture some essential aspect of the system, which are explored in the following section.

### 3.3 Exploring modifications of surface and surrounding

Silver nanoparticles are known to tarnish when exposed to air, even under ambient conditions.^44–46^ The tarnish layer formed on the surface – as for bulk silver – is typically identified as silver sulfide (Ag_2_S), resulting from atmospheric traces of *e.g.* hydrogen sulfide or carbonyl sulfide. Furthermore, silver can oxidize to form Ag_2_O; although it is largely assumed that elemental oxygen is required,^47,48^ it has also been reported that Ag particles oxidize in air.^49^ It is therefore plausible that Ag_2_S or Ag_2_O are present on the cube surface. In order to investigate their effect on the optical properties, we model them as a shell of homogeneous thickness replacing an outer layer of Ag, as depicted in Fig. S5b of the ESI.† This is knowingly a simplified description, as the surface coverage is likely to be inhomogeneous. Indeed, the TEM images (Fig. 2 and S8–S10†) display some material adhered irregularly to the cube surface. We note that a tarnish layer would be thicker than the removed Ag, as it incorporates external atoms,^47^ thus increasing the size of the cube. However, this does not have a significant effect due to the weak size dependence we have seen in Fig. 4a. In the model, Ag_2_S has a refractive index of *n* = 3.08 and an absorption index of *k* = 0.45, as measured^50^ on a natural tarnish of 3.9 nm thickness at a wavelength of 500 nm. For Ag_2_O the values *n* = 2.5 and *k* = 0.11 are used, as reported^48^ for thin Ag films exposed to oxygen plasma at a wavelength of 680 nm. The dispersion of *n* and *k* for silver sulfide^50^ and oxide^47^ was disregarded to simplify the description, as the exact composition and crystalline form of the material forming the tarnish layer is not known.

The cross section spectra as a function of the thickness of the sulfide (*t*_s_) or oxide (*t*_o_) shell are shown in Fig. 5g and h respectively, in anisole under normal incidence illumination. The effect on the D and Q peaks is reported in Table 1, and is much larger in comparison to all previously studied parameters, particularly for the Q peak. Specifically, the resonances red-shift due to the increased *n* at the surface, and at the same time their amplitude decreases due to the screening by *n* and the absorption *k* of the shell. The decrease of Ag volume as it is converted into Ag_2_S or Ag_2_O also contributes to reducing the scattering, but this is not the dominating factor, as indicated by the larger reduction produced by Ag_2_S (which has a larger *n* and *k*) with respect to Ag_2_O for equal thickness. Similar trends were already observed experimentally under induced sulfidation of silver cube ensembles in a sulfurous aqueous solution.^51^ As shown in Fig. 4b and c, these changes go precisely in the direction needed to move the simulated data towards the experiment. For instance, a sulfide shell as thin 0.5 nm, corresponding to a few atomic layers, would already result in a good match for many cubes. Such small thicknesses would be hard to identify by TEM without a specific elemental analysis. After adding a thin shell to match *λ*_D_ and *λ*_Q_ for each cube, the magnitude of *σ*_sca_ agrees on average well within the aforementioned ∼10% systematic uncertainty.

**Fig. 6:**
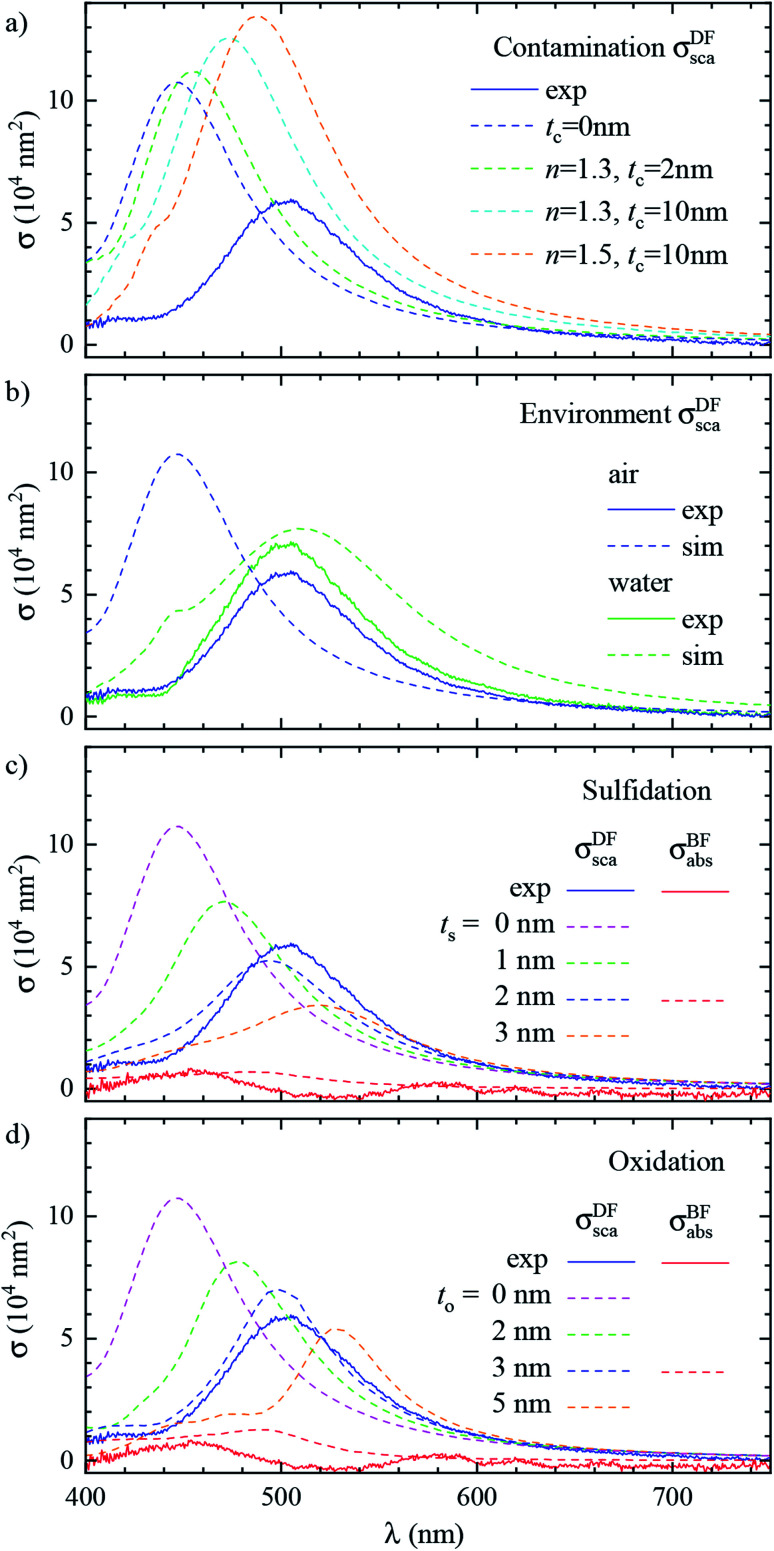
Effect of the local environment and surface composition. The measured (solid lines) and simulated (dashed lines) scattering spectrum *σ*^DF^_sca_(*λ*) in air of the same cube as Fig. 3 (#4) is shown. The model includes: (a) a layer of dielectric contaminant on the cube surface, with thickness *t* and refractive index *n* according to the legend; (b) a thick water (*n* = 1.33) film covering the sample; (c) a sulfide shell of thickness *t*_s_ having *n* = 3.08 and *k* = 0.45 replacing a superficial layer of silver; (d) an oxide shell of thickness *t*_o_ having *n* = 2.5 and *k* = 0.1 replacing a superficial layer of silver. Panel (b) also displays the experimental spectrum corrected with the scattering parameters computed in water (blue dashed line). Panel (c) and (d) also display the measured and simulated absorption spectra *σ*^BF^_abs_(*λ*) for the layer thicknesses best reproducing the scattering.

Let us now revisit the red shift of the D peak in air with respect to simulations, as can be seen in Fig. 3b and 4d. Such red shifts of 50 to 100 nm with respect to simulations were previously reported correlating TEM with quantitative optical (SMS) measurements of single gold rods^52^ and bipyramids^53^ in air. They were ascribed to the presence of surfactant residuals and/or thick water layers, and reproduced by simulations using a refractive index of the immersion medium up to 1.4. Recently, SMS on single gold bipyramids immobilized on glass substrates^54^ showed a red shift of the LSPR when changing the immersion medium from air to water, in contradiction with the water layer hypothesis. To verify whether the presence of organic contaminants could explain the measured shift in our case, we performed simulations with a dielectric thin shell encapsulating the cube; for details see Fig. S5c in the ESI.† The resulting scattering spectra for various shell properties are shown in Fig. 6a, and indeed display a sizeable red shift, but also an increased peak magnitude, which is incompatible with the experimental data. Furthermore, to match the experimental peak position a rather thick and optically dense shell would be required, but this is not visible in TEM images; see Fig. 3b. We note that from these results we can also estimate the effect of the PVP layer. The manufacturer does not specify the PVP thickness, and in ref. 55 a thickness of 1.5 nm was used. A PVP layer of more than 3 nm would be clearly visible in the acquired TEM. The refractive index of PVP is similar to the anisole immersion medium, so that the PVP layer has no significant influence on the data for anisole immersion. We have simulated with and without PVP in anisole and found a less than 1% change. For the data in air, PVP has some influence, comparable to the simulation of the contamination layer in Fig. 6a. An increase and red shift of the D peak is observed. For a 2 nm PVP layer, the effect is a fifth of the 10 nm *n* = 1.5 simulation, providing a 10 nm redshift of *λ*_LSPR_ and a 5% increase of *σ*^DF^_sca_.

Alternatively, the presence of a water layer – which is volatile and would not show up in TEM – could explain the red shift of measurements in air. Even though the samples were dried under a nitrogen flow, the ambient humidity might be sufficient to lead to the formation of such a layer. We simulated a thick water layer on the sample with a *n* = 1.33 immersion medium. As shown in Fig. 6b, the simulated D peak red shifts and decreases, reproducing the position and magnitude of the experiment, which was reanalysed using the scattering parameters for water immersion. However, the linewidth in water (150 nm) is much larger than the measured one (86 nm), due to a stronger radiative damping.^40^

Having ruled out organic contaminants and water layers as possible causes of the observed red shift, let us examine the silver sulfide or oxide shell hypothesis, which already proved capable of matching the simulation to the experiment in anisole. *σ*^DF^_sca_(*λ*) in air for various shell thicknesses is shown in Fig. 6c for Ag_2_S and in Fig. 6d for Ag_2_O. Similar to the anisole case, sulfidation or oxidation red-shifts the resonances and reduces their magnitude. Specifically, a 2 nm (3 nm) thick layer of Ag_2_S (Ag_2_O) matches the experimental D peak position, yielding at the same time a good agreement in magnitude and linewidth. Notably, the simulated absorption spectra *σ*^BF^_abs_(*λ*), for these thicknesses, shown in Fig. 6c and d, are still much smaller than the scattering, and consistent with the measurements, despite the absorption of the tarnish layers.

It is interesting to compare the layer thicknesses found in air to the smaller values required to match the spectra in anisole: about 0.5 nm Ag_2_S or 1 nm Ag_2_O; see Fig. 4. Notably, after the anisole immersion, the samples were dried in air and then stored in nitrogen at 4 °C until the measurements in air a few days later (see Fig. 1), potentially allowing for more tarnish or oxide to form. Indeed, a corrosion rate in air of the order of 1 nm per day has been reported previously.^44–46^ Furthermore, photo-oxidation of Ag cubes was reported^56^ under similar illumination conditions as in this work. However, more than 5 hours of illumination were required to produce a measurable change, while in our work spectra are taken over a few minutes, with total illumination times of each grid window below one hour. In the ESI Section S.VIII,† we analyze the correlation between the material adhered to the cubes seen in TEM and the cross-section spectra. We find an effective layer thickness around 2 nm, but no significant correlation between this thickness and the values of *λ*_LSPR_ and *σ*^DF^_sca_, as given in Fig. 4c. The formation of a thin tarnish layer is therefore a likely hypothesis explaining the experimental data. To verify this hypothesis, one could perform element-specific TEM such as electron energy loss or energy-dispersive X-ray spectroscopy.

## 4 Conclusions

This work describes a pipeline for correlative and quantitative optical and structural (TEM) characterization of an individual nano-particle. It combines a sample preparation protocol which allows correlating single-particle measurements in different environments, an optical microspectroscopy method to quantify the magnitude of *σ*_sca_ and *σ*_abs_, correlative TEM analysis, and comparison with accurate numerical simulations of the optical experiments. Let us emphasize that quantitative cross-section spectroscopy is far from routine characterisation, particularly for scattering, and in fact only a handful of such measurements can be found in the literature. To showcase the power of this approach we applied it to a technologically relevant particle system: silver cubes of 75 nm nominal size.

We highlighted the importance of reproducing accurately the high NA incoherent illumination employed in microscopy experiments, in order to quantitatively compare the simulation to experiment, through a refined modelling recipe. Notably, the simplified assumption of plane wave excitation is widespread in the literature of nanophotonics modelling. We show that this can be inadequate, since it not only affects the cross section magnitude, but also alters the overall appearance of the spectrum, as it fails to account for axially polarized components of the excitation, and their different coupling to resonant modes.

We performed a heuristic analysis, whereby various parameters of the simulated system are varied to establish their potential to match the experiment. While multiple relevant aspects (material properties, contamination, and degradation) carry a significant uncertainty even when the geometry is well characterized, the additional data gathered through quantitative and correlative measurements make the appraisal more stringent and revealing. In particular, we concluded that the observed deviations between the simulation and experiment cannot be substantially reduced by a tuning of the geometry within the bounds of the structural characterisation nor by the presence of organic contaminants, whereas a match is achieved by hypothesizing tarnishing or oxidation of a thin (0.5 to 3 nm) surface layer. This finding challenges a claim repeatedly found in the literature – based on the LSPR position, and not on its magnitude – that the red shift displayed by spectroscopy in air is due to organic contamination or water condensation. Notably, it shows that the additional information gained by the demonstrated quantitative correlative method can actually yield detailed knowledge at the single particle level, which would be difficult to attain otherwise.

The method can be applied to a wide range of particles, even in complex environments – in fact, any particle whose optical properties one is able to model numerically. Overall, the method pushes the boundaries of single particle characterization to a quantitative level which can prove instrumental for next-generation particle manufacturing and in improving nano-system designs.

## Author contribution

Y. W., P. B. and W. L. developed the workflow for correlative electron and optical microscopy. A. Z., P. B., and W. L. developed the quantitative optical microspectroscopy technique. Y. W. performed the sample preparation, optical microspectroscopy and electron microscopy. A. Z., Z. S., P. B. and W. L. developed the numerical model and methods. Z. S. and A. Z. performed the numerical simulations. All authors contributed to data interpretation and writing of the manuscript.

## Data availability

Information on the data created during this research, including how to access it, is available from the Cardiff University data archive at https://doi.org/10.17035/d.2019.0083359483.

## Conflicts of interest

There are no conflicts to declare.

## Supplementary Material

NA-002-D0NA00059K-s001
