# Supplementary material for: Quantitative optical microspectroscopy, electron microscopy, and modelling of individual silver nanocubes reveal surface compositional changes at the nanoscale
Source: Nanoscale Adv. 2020 Apr 22;2(6):2485–96. doi: 10.1039/d0na00059k (PMC9419171; doi:10.1039/d0na00059k)
Supplement: NA-002-D0NA00059K-s001 [file NA-002-D0NA00059K-s001.pdf]

Electronic Supplementary Material (ESI) for Nanoscale Advances.
This journal is © The Royal Society of Chemistry 2020

Supporting information to: Quantitative Optical Microspectroscopy, Electron Microscopy, and Modelling of Individual Silver Nanocubes Reveals Surface Compositional Changes at the Nanoscale

Yisu Wang,[1,*] Attilio Zilli,[1,2,*] Zoltan Sztranyovszky,[3]
Wolfgang Langbein,[3,†] and Paola Borri[1]

[1] *Cardiff University, School of Biosciences, Museum Avenue, Cardiff CF10 3AX, U.K.*
[2] *Present address: Politecnico di Milano, Department of Physics, Piazza Leonardo da Vinci 32, 20133 Milano, Italy*
[3] *Cardiff University, School of Physics and Astronomy, The Parade, Cardiff CF24 3AA, U.K.*

(Dated: April 18, 2020)

(29 pages, 11 figures, 1 table)

* These authors contributed in equal measure to this work
† langbeinww@cardiff.ac.uk

S.I. SAMPLE PREPARATION

Drop-casting is the most common and straightforward technique to immobilize nanoparticles (henceforth particles) on a grid suitable for transmission electron microscopy (TEM). However, drop-casting often leads to problems such as weak attachment, particle aggregation and deposition of debris from the solutes, all of which affect the correlation to TEM of the optical measurements. To overcome these problems, we developed a sample preparation method called *wet-casting* to immobilize metal particles onto a commercial TEM grid with a silica (SiO_2) membrane. Below we describe the wet-casting protocol we used for the silver nanocubes (henceforth cubes) to complement Sec. 2.1 of the paper. For further details the reader is referred to Ref. [S1].

Following Ref. [S2], the silica film was functionalized with 3-aminopropyl triethoxysilane (APTES) to enable binding of the silver cubes to the film. The grid was incubated with an etching solution made of 5 % (v/v) H_2SO_4 diluted into 30 % H_2O_2, in order to activate the silica surface by increasing the surface density of silanol groups. We used a 5 % concentration of H_2SO_4 (instead of the 30 % concentration commonly used) to ease the washing of the fragile grid. After mixing H_2O_2 and H_2SO_4, the temperature of the solution was cooled down to 65 °C (instead of the 90 °C temperature prescribed by the standard protocol) to avoid rupture and deformation of the silica film. The washed, acid-activated grid was then incubated in 1 % (v/v) of APTES in ethanol. One should always use fresh APTES (newly purchased or properly stored in a sealed anhydrous condition), because APTES can readily polymerize upon contact with water, forming large aggregates. Since the refractive index of APTES and anisole are similar, such aggregates are hardly noticeable during optical measurements with anisole immersion, but they are highly detrimental to the optical measurements in air, and to the subsequent TEM imaging. Additionally, to remove residual aggregates before use, the APTES stock was centrifuged at high speed (20k relative centrifugal force for 10 min) to precipitate the debris before dissolving it in ethanol for functionalization.

Fig. S1 shows that the wet-casting protocol yields a rather homogenous particle density on the silica surface. The cubes are stably immobilized onto the film via APTES functionalization, so that their positions are the same after optical measurement and TEM. Thanks to such strong particle attachment, the sample grid can be washed with deionized water and organic solvents such as anisole, ethanol and acetone, which allows us to correlate TEM imaging and optical measurement in different environments. In contrast, we found that wet-casting without APTES functionalization leads to particles being removed by anisole immersion. Both the dark-field images (Fig. 1) and the TEM images (Fig. 2) reported in the paper show a film surface largely free from debris. Moreover, the sample grid was stable in TEM at various projection angles, indicating there are no decomposing organic compounds deposited on the film surface. In contrast, we found that drop casting and drying results in the deposition of debris, which can cover the cubes and influence their optical properties in an ill-defined manner. After TEM characterization, dark circles can be seen around the imaged particles,

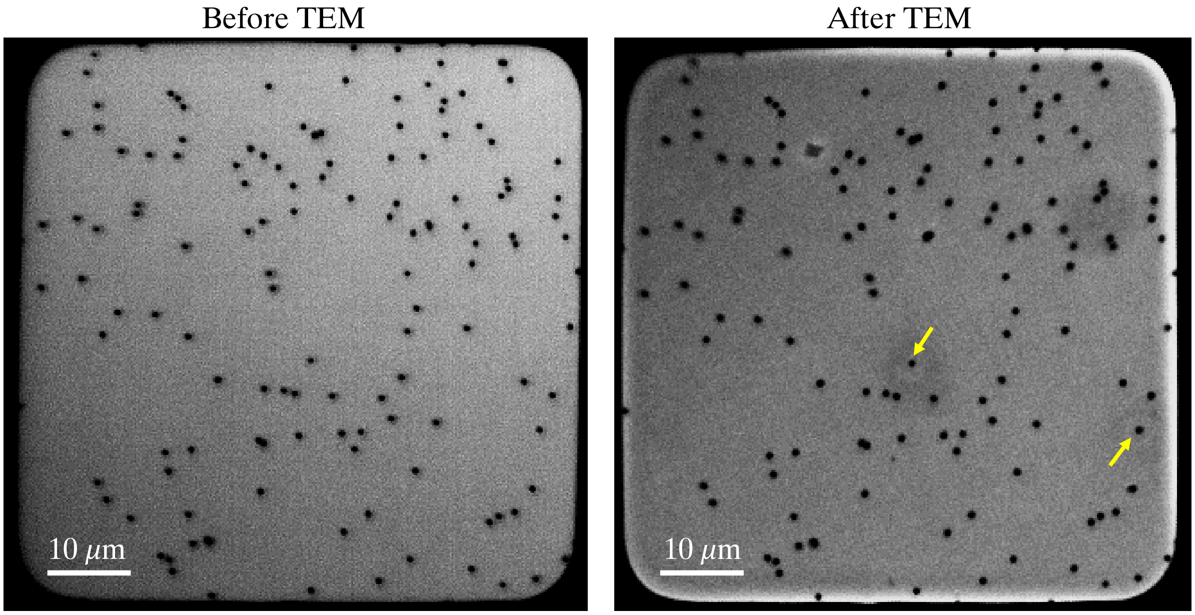

Figure S1. Optical transmission images of 75 nm edge silver cubes wet-cast on a silicon-supported 40 nm SiO_2 / 200 nm Si_3N_4 TEM grid (Ted Pella, Inc. 21530-10) functionalized with APTES. In the visible 50 µm sized windows the Si_3N_4 layer is removed, leaving the SiO_2 membrane exposed. Optical transmission micrographs were taken before (left) and after (right) TEM imaging. The arrows indicate particles imaged with TEM.

as highlighted by the arrows in Fig. S1. We ascribe these to damage of the SiO_2 membrane upon the prolonged exposure to the intense (200 kV) electron beam. On the other hand, we did not notice morphological changes of the cubes during TEM imaging, see Ref. [S1].

Optical measurements require that single particles are separated by few times the optical resolution, that is, few µm in the visible range. On the other hand, to identify the cubes in TEM imaging, a magnification of at least 2500 is required, corresponding to a field of view of at most $5 \times 5\,\mu m^2$, within which several cubes should be visible to recognize a configuration pattern. These two requirements are fulfilled for particle densities between 0.01 and $0.2\,\mu m^{-2}$. We are able to control the particle density by varying the concentration of the incubation solution, as shown in Fig. S2. The density was assessed by counting the bright spots in a dark-field (DF) scattering image. We note that most spots are green to yellow with a medium brightness, which are found in TEM to be single isolated cubes, while the occasionally observed red spots are found to be bipyramids, seemingly contained in the cube solution.

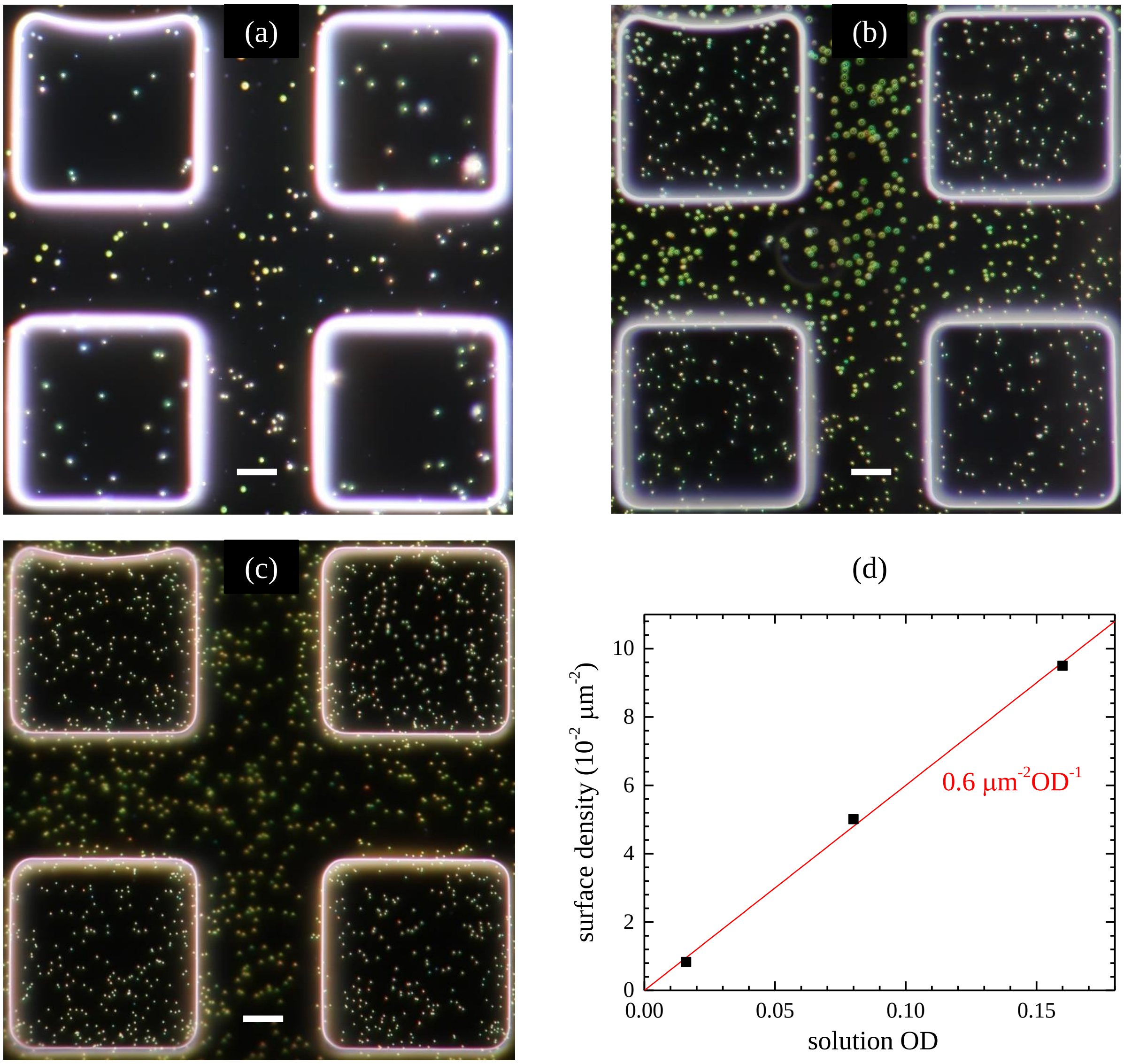

Figure S2. Panels (a)–(c): DF microscopy images in anisole of the sample grids, prepared by wet-casting 9 µl of suspension with various concentrations of 75 nm silver cubes in water. Scale bars are 10 µm. The images contain four SiO_2 (silica) square windows of 50 µm side length, supported by a Si_3N_4 mesh. The wet-cast solution concentrations are quantified using the optical density (OD) of the suspension for 1 cm path length at $\lambda = 515$ nm, and the conversion factor 3.6×10^8 cubes per mL and OD stated in the manufacturer data sheet. (a) 0.016 OD, corresponding to a concentration of 5.8×10^6 ml^{-1}; (b) 0.08 OD and 2.9×10^7 ml^{-1}; (c) 0.16 OD and 5.8×10^7 ml^{-1}. The number of particles in each silica window of 2.5×10^3 µm^2 area were counted using the find maxima plug-in of the software ImageJ, resulting in surface densities of (a) 8.3×10^{-3} µm^{-2}; (b) 50.1×10^{-3} µm^{-2}; (c) 95.0×10^{-3} µm^{-2}. These values are plotted in panel (d), and highlight a linear scaling of 0.6 /OD/µm^2 from optical to surface density.

S.II. EXCITING ELECTRIC FIELD IN A THREE-LAYERED MEDIUM

As discussed in the paper, numerical simulations play a twofold role in this work: They are instrumental to the quantitation of the optical cross sections, as well as serve as a reference for comparing quantitative measurements against. In sections S.II and S.III we provide details of the model we use to simulate absorption and scattering of a single nano-object under plane wave (PW) illumination. In sections S.IV to S.VI we discuss how multiple PW simulations can be combined analytically to model accurately the high-numerical aperture, incoherent illumination of our microscopy experiments.

We solve the frequency domain formulation of the electromagnetic problem using COMSOL Multiphysics®, a commercial software implementing the finite element method. The scattered field formalism commonly adopted for scattering problems relies on the decomposition of the total electric field into an exciting and a scattered component: $\boldsymbol{E}_{\rm tot} = \boldsymbol{E}_{\rm exc} + \boldsymbol{E}_{\rm sca}$. The solver computes $\boldsymbol{E}_{\rm sca}$ given $\boldsymbol{E}_{\rm exc}$, which is defined as the field in the absence of the scatterer. It is common modeling practice to compute $\boldsymbol{E}_{\rm exc}$ through a preliminary solving step where the particle volume is replaced by the surrounding medium, except for the simplest case of a PW propagating through a homogeneous medium. In view of the large number ($\sim 10^5$) of simulations required by our analysis, it is more expedient to input the electric field via an analytic expression. We already used this approach in a previous work [S3] to simulate the optical cross section of particles deposited on an optically-thick substrate, and observed a speed up by a factor 5 to 10 on comparison to the two-step solving scheme. In the experiments presented in this work, the particles are placed on a TEM grid with silica windows of 40 nm thickness, and therefore the simulation volume includes two planar interfaces, namely both sides of the silica film. Below we derive the analytic expression of $\boldsymbol{E}_{\rm exc}$ in such a three-layered dielectric medium illuminated by a PW having an arbitrary direction of incidence.

A. Formulation in spherical coordinates

The geometry considered and the notation adopted are summarized by Fig. S3. The problem can be approached by expressing the electric field in each medium as the sum of a transmitted wave propagating forward (subscript f) and a reflected wave propagating backward (subscript b)

$$\boldsymbol{E}_1 = \boldsymbol{E}_{1,\rm f,s} + \boldsymbol{E}_{1,\rm b,s} + \boldsymbol{E}_{1,\rm f,p} + \boldsymbol{E}_{1,\rm b,p}, \tag{S1-1}$$

$$\boldsymbol{E}_2 = \boldsymbol{E}_{2,\rm f,s} + \boldsymbol{E}_{2,\rm b,s} + \boldsymbol{E}_{2,\rm f,p} + \boldsymbol{E}_{2,\rm b,p}, \tag{S1-2}$$

$$\boldsymbol{E}_3 = \boldsymbol{E}_{3,\rm f,s} + \boldsymbol{E}_{3,\rm f,p}. \tag{S1-3}$$

The subscripts p and s denote the field components parallel and perpendicular to the plane of incidence, which is identified by the wavevector and the normal to the interfaces. Due to

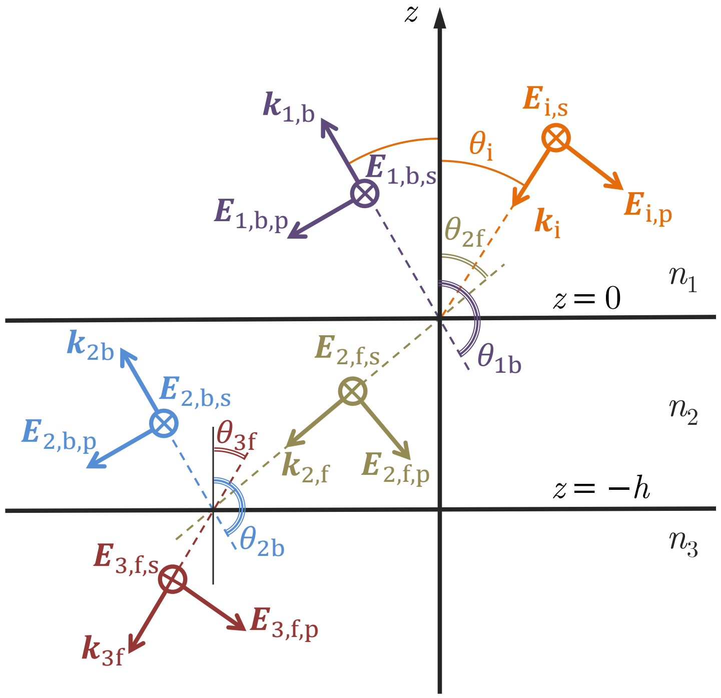

Figure S3. Sketch of the simulated geometry in the absence of the scatterer. Three dielectric media of refractive indices n_1, n_2, and n_3 are separated by two parallel planar interfaces at $z = 0$ and $z = -h$. Illumination comes from medium 1 and collection occurs in medium 3, while medium 2 represents the optically thin ($h = 40\,\mathrm{nm} \ll \lambda$) particle substrate. The subscripts f denotes forward propagation for the incident wave in medium 1 and the transmitted one in mediums 2 and 3, while the subscript b denotes backward propagation for the reflected wave in mediums 1 and 2. The subscript i, standing for incident, is a shorthand for 1,f. The plane of incidence $\varphi = \varphi_\mathrm{i}$ is shown, so that the p and s components of the electric field component (which are not mixed by the interfaces) are respectively parallel and perpendicular to the plane of the page.

the planar symmetry of the problem, the p and s components are not mixed upon reflection or transmission, and thus form a convenient basis to decompose the fields.

In order to write down the expression of all the terms in Eq. (S1), we need to refer to a coordinate system, and spherical coordinates (r, θ, φ) are an appropriate choice for the problem at hand. To understand the reason, let us consider the spherical unit vectors written in Cartesian components

$$\widehat{\boldsymbol{r}} = \begin{bmatrix} \sin\theta\cos\varphi \\ \sin\theta\sin\varphi \\ \cos\theta \end{bmatrix}, \quad \widehat{\boldsymbol{\theta}} = \begin{bmatrix} \cos\theta\cos\varphi \\ \cos\theta\sin\varphi \\ -\sin\theta \end{bmatrix}, \quad \widehat{\boldsymbol{\varphi}} = \begin{bmatrix} -\sin\varphi \\ \cos\varphi \\ 0 \end{bmatrix}. \tag{S2}$$

$\widehat{\boldsymbol{\theta}}$ and $\widehat{\boldsymbol{\varphi}}$ are respectively parallel and perpendicular with respect to a generic plane of incidence, namely a plane of constant φ. They thus coincide with the unit vectors $\widehat{\boldsymbol{p}}$ and $\widehat{\boldsymbol{s}}$ defining the orientation of the p and s field components. The observation that the PW

propagates in a direction perpendicular to both $\hat{\boldsymbol{p}}$ and $\hat{\boldsymbol{s}}$ completes the identification of the two vector triads: $(\hat{\boldsymbol{k}}, \hat{\boldsymbol{p}}, \hat{\boldsymbol{s}}) = (-\hat{\boldsymbol{r}}, \hat{\boldsymbol{\theta}}, \hat{\boldsymbol{\varphi}})$.

Therefore the field components in Eq. (S1) have the following expression in spherical coordinates

$$
\begin{aligned}
\boldsymbol{E}_{1,\mathrm{f,s}} &= E_{\mathrm{is}}\, \hat{\boldsymbol{\varphi}}(\varphi_\mathrm{i}) \exp[-in_1k_0\hat{\boldsymbol{r}}(\theta_\mathrm{i},\varphi_\mathrm{i})\cdot\boldsymbol{r}],\\
\boldsymbol{E}_{1,\mathrm{b,s}} &= E_{1\mathrm{bs}}(\theta_\mathrm{i})\, \hat{\boldsymbol{\varphi}}(\varphi_\mathrm{i}) \exp[-in_1k_0\hat{\boldsymbol{r}}(\pi-\theta_\mathrm{i},\varphi_\mathrm{i})\cdot\boldsymbol{r}],\\
\boldsymbol{E}_{1,\mathrm{f,p}} &= E_{\mathrm{ip}}\, \hat{\boldsymbol{\theta}}(\theta_\mathrm{i},\varphi_\mathrm{i}) \exp[-in_1k_0\hat{\boldsymbol{r}}(\theta_\mathrm{i},\varphi_\mathrm{i})\cdot\boldsymbol{r}],\\
\boldsymbol{E}_{1,\mathrm{b,p}} &= E_{1\mathrm{bp}}(\theta_\mathrm{i})\, \hat{\boldsymbol{\theta}}(\pi-\theta_\mathrm{i},\varphi_\mathrm{i}) \exp[-in_1k_0\hat{\boldsymbol{r}}(\pi-\theta_\mathrm{i},\varphi_\mathrm{i})\cdot\boldsymbol{r}],\\
\boldsymbol{E}_{2,\mathrm{f,s}} &= E_{2\mathrm{fs}}(\theta_\mathrm{i})\, \hat{\boldsymbol{\varphi}}(\varphi_\mathrm{i}) \exp[-in_2k_0\hat{\boldsymbol{r}}(\theta_{2\mathrm{f}},\varphi_\mathrm{i})\cdot\boldsymbol{r}],\\
\boldsymbol{E}_{2,\mathrm{b,s}} &= E_{2\mathrm{bs}}(\theta_\mathrm{i})\, \hat{\boldsymbol{\varphi}}(\varphi_\mathrm{i}) \exp[-in_2k_0\hat{\boldsymbol{r}}(\pi-\theta_{2\mathrm{f}},\varphi_\mathrm{i})\cdot\boldsymbol{r}],\\
\boldsymbol{E}_{2,\mathrm{f,p}} &= E_{2\mathrm{fp}}(\theta_\mathrm{i})\, \hat{\boldsymbol{\theta}}(\theta_{2\mathrm{f}},\varphi_\mathrm{i}) \exp[-in_2k_0\hat{\boldsymbol{r}}(\theta_{2\mathrm{f}},\varphi_\mathrm{i})\cdot\boldsymbol{r}],\\
\boldsymbol{E}_{2,\mathrm{b,p}} &= E_{2\mathrm{bp}}(\theta_\mathrm{i})\, \hat{\boldsymbol{\theta}}(\pi-\theta_{2\mathrm{f}},\varphi_\mathrm{i}) \exp[-in_2k_0\hat{\boldsymbol{r}}(\pi-\theta_{2\mathrm{f}},\varphi_\mathrm{i})\cdot\boldsymbol{r}],\\
\boldsymbol{E}_{3,\mathrm{f,s}} &= E_{3\mathrm{fs}}(\theta_\mathrm{i})\, \hat{\boldsymbol{\varphi}}(\varphi_\mathrm{i}) \exp[-in_3k_0\hat{\boldsymbol{r}}(\theta_{3\mathrm{f}},\varphi_\mathrm{i})\cdot\boldsymbol{r}],\\
\boldsymbol{E}_{3,\mathrm{f,p}} &= E_{3\mathrm{fp}}(\theta_\mathrm{i})\, \hat{\boldsymbol{\theta}}(\theta_{3\mathrm{f}},\varphi_\mathrm{i}) \exp[-in_3k_0\hat{\boldsymbol{r}}(\theta_{3\mathrm{f}},\varphi_\mathrm{i})\cdot\boldsymbol{r}],
\end{aligned}
\tag{S3}
$$

where $k_0 = 2\pi/\lambda_0$ is the magnitude of the wavevector in vacuum. Incidentally, Comsol adopts the electrical engineering sign convention of plane waves $\exp\left[-i(\boldsymbol{k}\cdot\boldsymbol{r}-\omega t)\right]$, which is complex conjugated to what commonly used in optics and in this work; therefore, (S3) must be conjugated for using in Comsol. The subscript i, standing for incident, has been introduced as a shorthand for 1f. Note that all the polar angles appearing in Eq. (S3) are functions of the polar angle of incidence θ_i alone, thanks to Snell's law

$$
\begin{aligned}
\theta_{2\mathrm{f}} &= \arcsin\left(\frac{n_1}{n_2}\sin\theta_\mathrm{i}\right),\\
\theta_{3\mathrm{f}} &= \arcsin\left(\frac{n_1}{n_3}\sin\theta_\mathrm{i}\right).
\end{aligned}
\tag{S4}
$$

The planar character of the problem implies that the azimuth of propagation φ_i is the same in all media, and that the field amplitudes appearing in (S3) are independent from φ_i. This enables us compute them for any given plane of incidence. For the sake of simplicity, let us then assume without loss of generality $\varphi_\mathrm{i} = 0$, which entails $k_y = 0$.

Finally, we emphasize that the formalism just established is valid as well in presence of evanescent waves (that is, non-propagating modes), for which θ is complex and k_z becomes purely imaginary, leading to an exponential decay of the field amplitude away from the interface.

B. Computation of the amplitudes

In this section we compute the 8 unknown amplitudes of the electric field terms listed in Eq. (S3). This is achieved by imposing the appropriate conditions of continuity of the electric and magnetic field at interfaces between dielectric layers. As already mentioned, the planar symmetry of the problem enables us to treat separately the p and s component of the electric field. To unburden the notation, in each subsection the relative polarization subscript is dropped.

1. *Electric field polarized parallel to the plane of incidence (E_p)*

We begin by decomposing the electric field into its components parallel ($\|$) and perpendicular ($\perp$) to the interfaces, which lie along the Cartesian axes $\widehat{\boldsymbol{x}}$ and $\widehat{\boldsymbol{z}}$. The magnetic field is obtained via the relation $\boldsymbol{H} = \frac{n}{\mu_0 c}\widehat{\boldsymbol{k}} \times \boldsymbol{E}$, where we replaced μ with μ_0 since we deal with non-magnetic media The fields in media 1, 2, and 3 read

$$\boldsymbol{E}_1 = \widehat{\boldsymbol{x}} \cos\theta_\mathrm{i} \left(\mathcal{E}_{1\mathrm{b}} - \mathcal{E}_{1\mathrm{f}}\right) - \widehat{\boldsymbol{z}} \sin\theta_\mathrm{i} \left(\mathcal{E}_{1\mathrm{b}} + \mathcal{E}_{1\mathrm{f}}\right), \tag{S5-E1}$$

$$\boldsymbol{H}_1 = \widehat{\boldsymbol{y}} \frac{n_1}{c\mu_0} \left(\mathcal{E}_{1\mathrm{b}} + \mathcal{E}_{1\mathrm{f}}\right), \tag{S5-H1}$$

$$\boldsymbol{E}_2 = \widehat{\boldsymbol{x}} \cos\theta_{2\mathrm{f}} \left(\mathcal{E}_{2\mathrm{b}} - \mathcal{E}_{2\mathrm{f}}\right) - \widehat{\boldsymbol{z}} \sin\theta_{2\mathrm{f}} \left(\mathcal{E}_{2\mathrm{b}} + \mathcal{E}_{2\mathrm{f}}\right), \tag{S5-E2}$$

$$\boldsymbol{H}_2 = \widehat{\boldsymbol{y}} \frac{n_2}{c\mu_0} \left(\mathcal{E}_{2\mathrm{b}} + \mathcal{E}_{2\mathrm{f}}\right), \tag{S5-H2}$$

$$\boldsymbol{E}_3 = -\widehat{\boldsymbol{x}} \cos\theta_{3\mathrm{f}}\, \mathcal{E}_{3\mathrm{f}} - \widehat{\boldsymbol{z}} \sin\theta_{3\mathrm{f}}\, \mathcal{E}_{3\mathrm{f}}, \tag{S5-E3}$$

$$\boldsymbol{H}_3 = \widehat{\boldsymbol{y}} \frac{n_3}{c\mu_0} \mathcal{E}_{3\mathrm{f}}. \tag{S5-H3}$$

where $\mathcal{E}_. = E_. \exp(i\boldsymbol{k}_. \cdot \boldsymbol{r})$. Imposing that $\boldsymbol{E}_\|$ is continuous at the interface $z = 0$ gives

$$-\cos\theta_\mathrm{i} E_\mathrm{i}\, e^{ik_{\mathrm{i}x}x} + \cos\theta_\mathrm{i} E_{1\mathrm{b}}\, e^{ik_{1\mathrm{b}x}x} = -\cos\theta_{2\mathrm{f}} E_{2\mathrm{f}}\, e^{ik_{2\mathrm{f}x}x} + \cos\theta_{2\mathrm{f}} E_{2\mathrm{b}}\, e^{ik_{2\mathrm{b}x}x}. \tag{S6}$$

Due to the translational symmetry of the problem along the interface, Eq. (S6) must hold for every value of x, requiring $k_{\mathrm{i}x} = k_{1\mathrm{b}x} = k_{2\mathrm{f}x} = k_{2\mathrm{b}x}$. The oscillatory terms thereby cancel out and we are left with

$$-\cos\theta_\mathrm{i} E_\mathrm{i} + \cos\theta_\mathrm{i} E_{1\mathrm{b}} = -\cos\theta_{2\mathrm{f}} E_{2\mathrm{f}} + \cos\theta_{2\mathrm{f}} E_{2\mathrm{b}}. \tag{S7}$$

Imposing that $\boldsymbol{H}_\|$ is continuous at the interface $z = 0$ gives

$$n_1 E_\mathrm{i} + n_1 E_{1\mathrm{b}} = n_2 E_{2\mathrm{f}} + n_2 E_{2\mathrm{b}}. \tag{S8}$$

Imposing that $\boldsymbol{E}_\parallel$ is continuous at the interface $z = -h$ gives

$$-\cos\theta_{2\mathrm{f}} E_{2\mathrm{f}}\, e^{-ik_{2\mathrm{f}z}h} + \cos\theta_{2\mathrm{f}} E_{2\mathrm{b}}\, e^{-ik_{2\mathrm{b}z}h} = -\cos\theta_{3\mathrm{f}} E_{3\mathrm{f}}\, e^{-ik_{3\mathrm{f}z}h}. \tag{S9}$$

Imposing that $\boldsymbol{H}_\parallel$ is continuous at the interface $z = -h$ gives

$$n_2 E_{2\mathrm{f}}\, e^{-ik_{2\mathrm{f}z}h} + n_2 E_{2\mathrm{b}}\, e^{-ik_{2\mathrm{b}z}h} = n_3 E_{3\mathrm{f}}\, e^{-ik_{3\mathrm{f}z}h}. \tag{S10}$$

Eq. (S9) and Eq. (S10) form a linear system in the unknowns $E_{2\mathrm{f}}$ and $E_{2\mathrm{b}}$. The solution obtained by subtracting or summing $n_2\times$(S9) to $\cos\theta_{2\mathrm{f}}\times$(S10) is

$$E_{2\mathrm{f}} = \frac{n_3\cos\theta_{2\mathrm{f}} + n_2\cos\theta_{3\mathrm{f}}}{2n_2\cos\theta_{2\mathrm{f}}} E_{3\mathrm{f}}\, e^{ik_{2\mathrm{f}z}h}\, e^{-ik_{3\mathrm{f}z}h}, \tag{S11-2f}$$

$$E_{2\mathrm{b}} = \frac{n_3\cos\theta_{2\mathrm{f}} - n_2\cos\theta_{3\mathrm{f}}}{2n_2\cos\theta_{2\mathrm{f}}} E_{3\mathrm{f}}\, e^{ik_{2\mathrm{b}z}h}\, e^{-ik_{3\mathrm{f}z}h}, \tag{S11-2b}$$

which relate the amplitude of the electric fields in medium 2 and 3.

We are finally in the position of expressing the amplitudes in medium 2 and 3 as a function of E_i. To simplify the expressions, let us define the coefficients $c_{ij} \equiv n_i\cos\theta_{j\mathrm{f}}$. By substituting the solution (S11) into Eq. (S7) and Eq. (S8) we obtain respectively

$$-E_\mathrm{i} + E_{1\mathrm{b}} = \frac{E_{3\mathrm{f}}\, e^{-ik_{3\mathrm{f}z}h}}{2c_{21}} \left[-(c_{32}+c_{23})e^{ik_{2\mathrm{f}z}h} + (c_{32}-c_{23})e^{ik_{2\mathrm{b}z}h}\right], \tag{S12}$$

$$+E_\mathrm{i} + E_{1\mathrm{b}} = \frac{E_{3\mathrm{f}}\, e^{-ik_{3\mathrm{f}z}h}}{2c_{12}} \left[+(c_{32}+c_{23})e^{ik_{2\mathrm{f}z}h} + (c_{32}-c_{23})e^{ik_{2\mathrm{b}z}h}\right]. \tag{S13}$$

Eq. (S12) and Eq. (S13) form a linear system in the unknowns $E_{1\mathrm{b}}$ and $E_{3\mathrm{f}}$ having the solution

$$\frac{E_{3\mathrm{f}}}{E_\mathrm{i}} = \frac{4c_{12}c_{21}\, e^{ik_{3\mathrm{f}z}h}}{(c_{32}+c_{23})(c_{12}+c_{21})e^{ik_{2\mathrm{f}z}h} + (c_{32}-c_{23})(c_{21}-c_{12})e^{ik_{2\mathrm{b}z}h}}, \tag{S14-3f}$$

$$\frac{E_{1\mathrm{b}}}{E_\mathrm{i}} = \frac{(c_{32}+c_{23})(c_{21}-c_{12})e^{ik_{2\mathrm{f}z}h} + (c_{32}-c_{23})(c_{21}+c_{12})e^{ik_{2\mathrm{b}z}h}}{(c_{32}+c_{23})(c_{21}+c_{12})e^{ik_{2\mathrm{f}z}h} + (c_{32}-c_{23})(c_{21}-c_{12})e^{ik_{2\mathrm{b}z}h}}. \tag{S14-1b}$$

The amplitudes in medium 2 are found by substituting Eq. (S14-3f) into Eq. (S11)

$$\frac{E_{2\mathrm{f}}}{E_\mathrm{i}} = \frac{2}{c_{22}} \frac{c_{12}c_{21}(c_{32}+c_{23})e^{ik_{2\mathrm{f}z}h}}{(c_{32}+c_{23})(c_{12}+c_{21})e^{ik_{2\mathrm{f}z}h} + (c_{32}-c_{23})(c_{21}-c_{12})e^{ik_{2\mathrm{b}z}h}}, \tag{S14-2f}$$

$$\frac{E_{2\mathrm{b}}}{E_\mathrm{i}} = \frac{2}{c_{22}} \frac{c_{12}c_{21}(c_{32}-c_{23})e^{ik_{2\mathrm{b}z}h}}{(c_{32}+c_{23})(c_{12}+c_{21})e^{ik_{2\mathrm{f}z}h} + (c_{32}-c_{23})(c_{21}-c_{12})e^{ik_{2\mathrm{b}z}h}}. \tag{S14-2b}$$

2. Electric field polarized perpendicular to the plane of incidence (E_s)

Analogously to Eq. (S5), fields in media 1, 2, and 3 can be decomposed into their components $\parallel$ and $\perp$ to the interfaces as

$$\boldsymbol{E}_1 = \widehat{\boldsymbol{y}}\left(\mathcal{E}_\mathrm{i} + \mathcal{E}_\mathrm{1b}\right), \tag{S15-E1}$$

$$\boldsymbol{H}_1 = \frac{n_1}{c\mu_0}\Big(\widehat{\boldsymbol{x}}\cos\theta_\mathrm{i}\left(\mathcal{E}_\mathrm{i} - \mathcal{E}_\mathrm{1b}\right) + \widehat{\boldsymbol{z}}\sin\theta_\mathrm{i}\left(\mathcal{E}_\mathrm{i} + \mathcal{E}_\mathrm{1b}\right)\Big), \tag{S15-H1}$$

$$\boldsymbol{E}_2 = \widehat{\boldsymbol{y}}\left(\mathcal{E}_\mathrm{2f} + \mathcal{E}_\mathrm{2b}\right), \tag{S15-E2}$$

$$\boldsymbol{H}_2 = \frac{n_2}{c\mu_0}\Big(\widehat{\boldsymbol{x}}\cos\theta_\mathrm{2f}\left(\mathcal{E}_\mathrm{2f} - \mathcal{E}_\mathrm{2b}\right) + \widehat{\boldsymbol{z}}\sin\theta_\mathrm{2f}\left(\mathcal{E}_\mathrm{2f} + \mathcal{E}_\mathrm{2b}\right)\Big), \tag{S15-H2}$$

$$\boldsymbol{E}_3 = \widehat{\boldsymbol{y}}\,\mathcal{E}_\mathrm{3f}. \tag{S15-E3}$$

$$\boldsymbol{H}_3 = \frac{n_3}{c\mu_0}\left(\widehat{\boldsymbol{x}}\cos\theta_\mathrm{3f}\mathcal{E}_\mathrm{3f} + \widehat{\boldsymbol{z}}\sin\theta_\mathrm{3f}\mathcal{E}_\mathrm{3f}\right), \tag{S15-H3}$$

Imposing that $\boldsymbol{E}_\parallel$ is continuous at the interface $z = 0$ gives

$$E_\mathrm{i}\, e^{ik_{\mathrm{i}x}x} + E_\mathrm{1b}\, e^{ik_{\mathrm{1b}x}x} = E_\mathrm{2f}\, e^{ik_{\mathrm{2f}x}x} + E_\mathrm{2b}\, e^{ik_{\mathrm{2b}x}x}. \tag{S16}$$

Like Eq. (S6), this relation must hold for every value of x, leading to $k_{\mathrm{i}x} = k_{\mathrm{1b}x} = k_{\mathrm{2f}x} = k_{\mathrm{2b}x}$. The oscillatory terms thereby cancel out and we are left with

$$E_\mathrm{i} + E_\mathrm{1b} = E_\mathrm{2f} + E_\mathrm{2b}. \tag{S17}$$

Imposing that $\boldsymbol{H}_\parallel$ is continuous at the interface $z = 0$ gives

$$n_1\cos\theta_i(E_\mathrm{i} - E_\mathrm{1b}) = n_2\cos\theta_\mathrm{2f}(E_\mathrm{2f} - E_\mathrm{2b}). \tag{S18}$$

Imposing that $\boldsymbol{E}_\parallel$ is continuous at the interface $z = -h$ gives

$$E_\mathrm{2f}\, e^{-ik_{\mathrm{2f}z}h} + E_\mathrm{2b}\, e^{-ik_{\mathrm{2b}z}h} = E_\mathrm{3f}\, e^{-ik_{\mathrm{3f}z}h}. \tag{S19}$$

Imposing that $\boldsymbol{H}_\parallel$ is continuous at the interface $z = -h$ gives

$$n_2\cos\theta_\mathrm{2f}\left(E_\mathrm{2f}\, e^{-ik_{\mathrm{2f}z}h} - E_\mathrm{2b}\, e^{-ik_{\mathrm{2b}z}h}\right) = n_3\cos\theta_\mathrm{3f}E_\mathrm{3f}\, e^{-ik_{\mathrm{3f}z}h}. \tag{S20}$$

Eq. (S19) and Eq. (S20) form a linear system in the unknowns $E_{2\mathrm{f}}$ and $E_{2\mathrm{b}}$. The solution obtained by summing or subtracting $n_2 \cos\theta_{2\mathrm{f}} \times$(S19) to (S20) is

$$E_{2\mathrm{f}} = \frac{E_{3\mathrm{f}}}{2}\, e^{i(k_{2\mathrm{f}z} - k_{3\mathrm{f}z})h} \left(1 + \frac{n_3 \cos\theta_{3\mathrm{f}}}{n_2 \cos\theta_{2\mathrm{f}}}\right) \tag{S21-2f}$$

$$E_{2\mathrm{b}} = \frac{E_{3\mathrm{f}}}{2}\, e^{i(k_{2\mathrm{b}z} - k_{3\mathrm{f}z})h} \left(1 - \frac{n_3 \cos\theta_{3\mathrm{f}}}{n_2 \cos\theta_{2\mathrm{f}}}\right) \tag{S21-2b}$$

which relate the amplitude of the electric fields in medium 2 and 3.

We are finally in the position of expressing the amplitudes in medium 2 and 3 as a function of E_i. By substituting the solution (S21) into Eq. (S17) and Eq. (S18) we obtain respectively

$$E_\mathrm{i} + E_{1\mathrm{b}} = \frac{E_{3\mathrm{f}}}{2}\, e^{-ik_{3\mathrm{f}z}h} \left[e^{ik_{2\mathrm{f}z}h} \left(1 + \frac{c_{33}}{c_{22}}\right) + e^{ik_{2\mathrm{b}z}h} \left(1 - \frac{c_{33}}{c_{22}}\right) \right], \tag{S22}$$

$$E_\mathrm{i} - E_{1\mathrm{b}} = \frac{c_{22}}{c_{11}} \frac{E_{3\mathrm{f}}}{2}\, e^{-ik_{3\mathrm{f}z}h} \left[e^{ik_{2\mathrm{f}z}h} \left(1 + \frac{c_{33}}{c_{22}}\right) - e^{ik_{2\mathrm{b}z}h} \left(1 - \frac{c_{33}}{c_{22}}\right) \right]. \tag{S23}$$

Eq. (S22) and Eq. (S23) form a linear system in the unknowns $E_{1\mathrm{b}}$ and $E_{3\mathrm{f}}$ having solution

$$\frac{E_{3\mathrm{f}}}{E_\mathrm{i}} = \frac{4c_{11}c_{22}e^{ik_{3\mathrm{f}z}h}}{(c_{22}+c_{33})(c_{11}+c_{22})e^{ik_{2\mathrm{f}z}h} + (c_{22}-c_{33})(c_{11}-c_{22})e^{ik_{2\mathrm{b}z}h}}, \tag{S24-3f}$$

$$\frac{E_{1\mathrm{b}}}{E_\mathrm{i}} = \frac{(c_{11}-c_{22})(c_{22}+c_{33})e^{ik_{2\mathrm{f}z}h} + (c_{11}+c_{22})(c_{22}-c_{33})e^{ik_{2\mathrm{b}z}h}}{(c_{11}+c_{22})(c_{22}+c_{33})e^{ik_{2\mathrm{f}z}h} + (c_{11}-c_{22})(c_{22}-c_{33})e^{ik_{2\mathrm{b}z}h}}. \tag{S24-1b}$$

The amplitudes in medium 2 are found by substituting Eq. (S24-3f) into Eq. (S21)

$$\frac{E_{2\mathrm{f}}}{E_\mathrm{i}} = \frac{2c_{11}(c_{22}+c_{33})e^{ik_{2\mathrm{f}z}h}}{(c_{22}+c_{33})(c_{11}+c_{22})e^{ik_{2\mathrm{f}z}h} + (c_{22}-c_{33})(c_{11}-c_{22})e^{ik_{2\mathrm{b}z}h}}, \tag{S24-2f}$$

$$\frac{E_{2\mathrm{b}}}{E_\mathrm{i}} = \frac{2c_{11}(c_{22}-c_{33})e^{ik_{2\mathrm{b}z}h}}{(c_{22}+c_{33})(c_{11}+c_{22})e^{ik_{2\mathrm{f}z}h} + (c_{22}-c_{33})(c_{11}-c_{22})e^{ik_{2\mathrm{b}z}h}}. \tag{S24-2b}$$

Table S1. TEM characterization of the geometry of the individual cubes used for modelling, as well as simulated and measured position λ_D and full width at half maximum Δ_D of the D resonance in anisole. Mean and standard deviation of each quantity across the set of cubes are also given. All values in units of nm.

Cube#	L_x	L_y	R_c	λ_D(exp)	λ_D(sim)	Δ_D(exp)	Δ_D(sim)
1	64.7	66.0	11.2	541	535	106	99
2	77.1	82.5	15.1	567	563	163	164
3	70.7	70.7	13.2	556	543	121	118
4	75.4	71.6	14.1	552	549	139	131
5	83.1	85.2	19.2	580	563	174	177
6	82.8	82.0	17.4	575	563	167	172
7	76.6	78.8	13.3	573	562	162	154
8	73.9	72.5	13.9	559	547	127	129
9	69.6	69.8	16.8	542	529	110	107
10	81.4	82.7	17.7	576	560	163	167
11	70.5	69.6	12.4	555	543	121	117
mean	75.1	75.6	14.9	561	551	141	139
stdev	5.9	6.7	2.5	14	12	25	28

S.III. MODEL GEOMETRY

Fig. S4 shows the model geometry with a typical mesh employed by the numerical solver. The geometry is built entirely in COMSOL by assembling primitive geometric shapes. The investigated "cubes" are represented as rectangular cuboids with unequal edge lengths (*i. e.* face-to-face distances) all close to the nominal 75 nm size. The edge lengths measured with TEM are reported in Table S1 and deviate within ±10 nm from the nominal value. The length L_z along the TEM axis cannot be assessed in our electron micrographs, and is taken as the arithmetic average of L_x and L_y. The cuboid edges and corners are rounded by cylinders and spheres, with radii of curvature R_c ranging from 11 to 19 nm, see Table S1. The simulation volume is approximately halved by a $h = 40$ nm thin dielectric slab perpendicular to $\boldsymbol{z}$ representing the silica membrane of the TEM grid. The slab partitions the immersion medium (anisole or air in our experiments) with refractive index $n_1 = n_3$. The cube lies flat on this membrane (on the $z > 0$ or $z < 0$ side for anisole or air respectively) near the center of the simulation volume. The simulation volume is a sphere large enough for its boundaries to be at least $\lambda/(2n_1)$ away from the cube surface — enough to put them outside the so-called reactive near-field region of the scatterer. The simulated volume is encircled by a perfectly matched layer (PML) of homogeneous thickness $\lambda/(2n_1)$ which absorbs efficiently the scattered light, thereby acting as an open boundary for radiation and mimicking an infinite simulation space.

We refined the user-controlled mesh until the optical cross section values converged within

a 1 % tolerance. This ensures that the simulation results are sufficiently independent of meshing, so that the dominant source of uncertainty is the limited knowledge of the system properties (geometry and material specifications). Specifically, such convergence occurs for a maximum mesh element size of 14 nm in the nano-object volume. In each medium the element size is capped to $\lambda/n/5$ to ensure a fine spatial sampling of the electromagnetic field. The PML is meshed with 5 elements in the radial direction, as is recommended practice for this kind of simulations. With the meshing described, the simulations have approximately 3×10^5 degrees of freedom and solve in about 15 s (including all post-processing) on a modern workstation (Intel Core i7-5830K CPU).

In the paper we consider several superficial layers wrapping the silver core of the cube; namely, a surfactant, a sulfidation or oxidation, and a contamination layer. Fig. S5 depicts the geometries corresponding to the various simulated configurations, and in particular the relative disposition of the layers when more than one are present. The layers are modeled with a homogeneous thickness, except for the contamination layer, which is absent on the face in contact with the silica membrane, see panel c. The surfactant is always present and has a fixed thickness $t = 2$ nm. The sulfidation or oxidation layer derives from a chemical modification of silver; it is therefore placed within the surfactant shell, inside the cube size L. In contrast, the contamination layer is placed on the outer side of the surfactant, and its thickness is not included in the cube size.

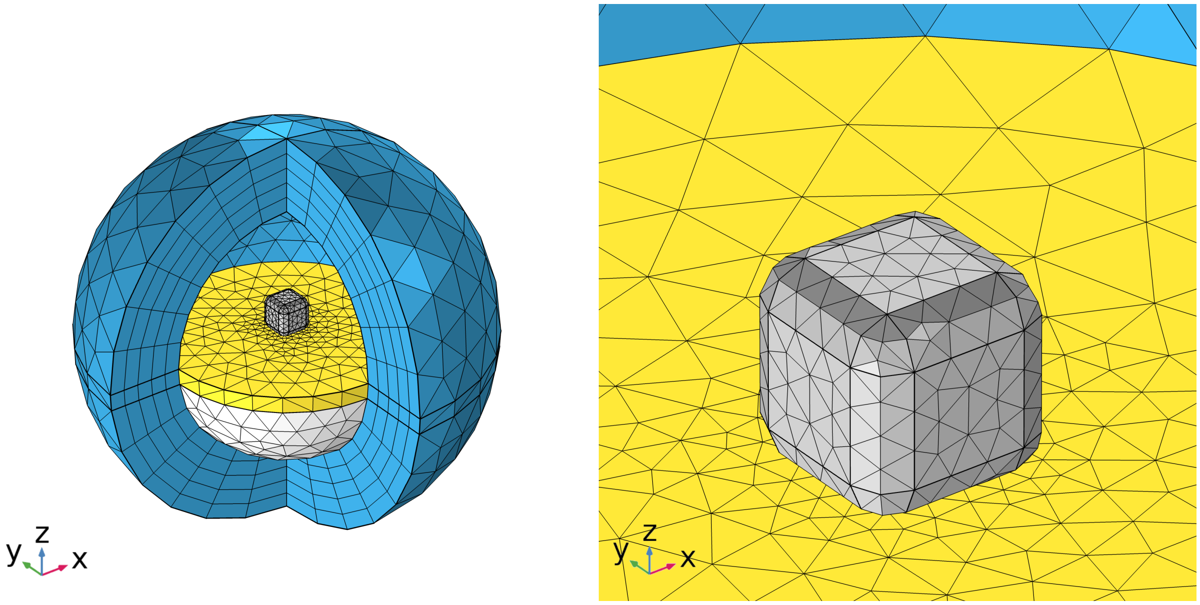

Figure S4. Model geometry with a typical mesh employed by the numerical solver. Left: Global view; right: close-up on the cube. The PML is colored in blue, the silica membrane in yellow, the immersion medium in white below the membrane and hidden above to show the cube, in gray, of $L = 75$ nm edge length and $R_c = 15$ nm edge rounding. A detailed description is given in the text.

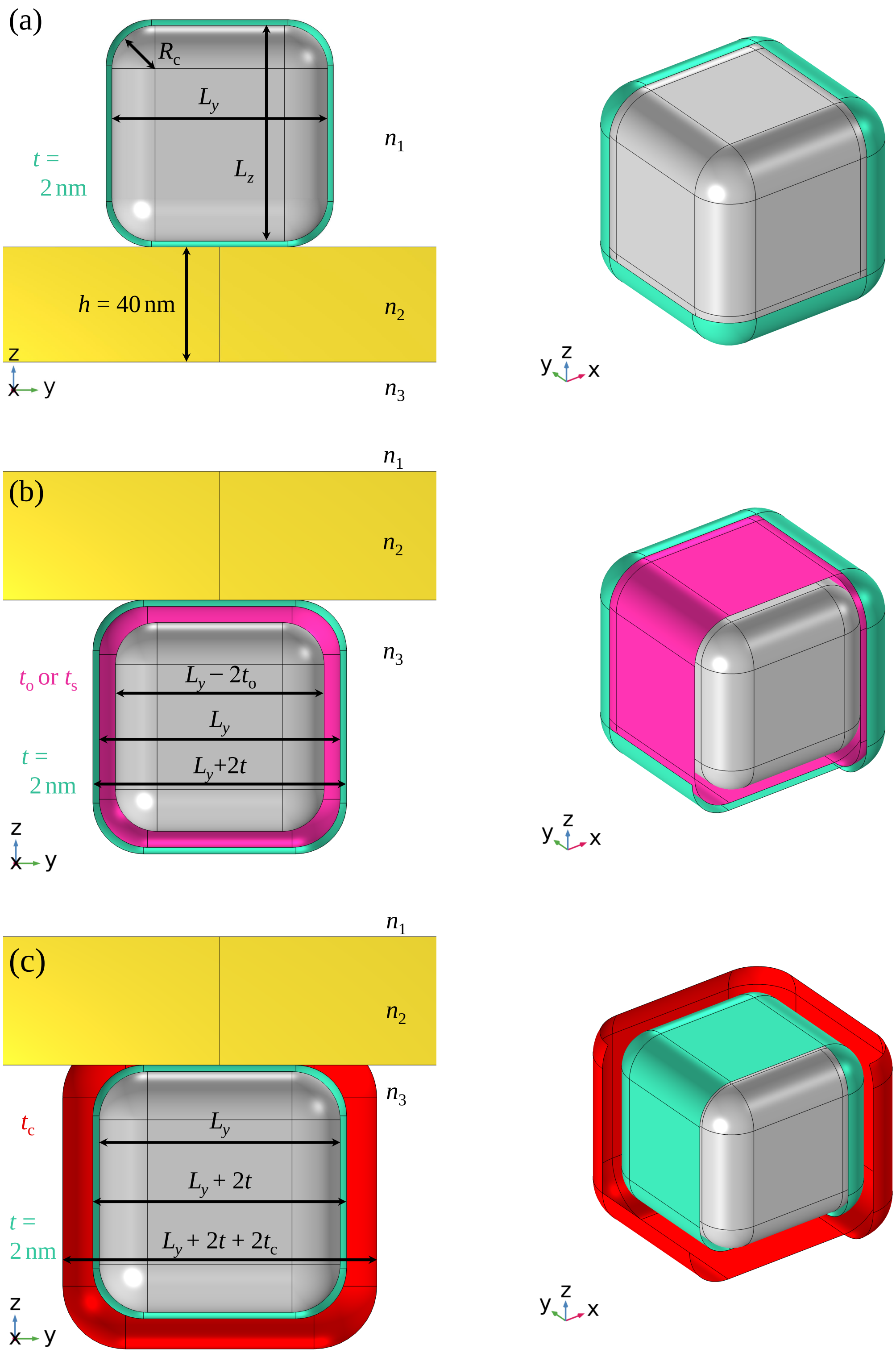

Figure S5. Geometry of the nano-object used for numerical modeling. Left: Side view; right: off-axis view. The colors indicate different materials. White: immersion medium (air or anisole); yellow: silica; grey: silver; pink: silver sulfide or oxide; green: surfactant (PVP); red: organic contaminant. Panel (a) depicts the configuration used for measurements in anisole (with the cube placed above the silica membrane), whereas (b) and (c) depict the configuration used for measurements in air.

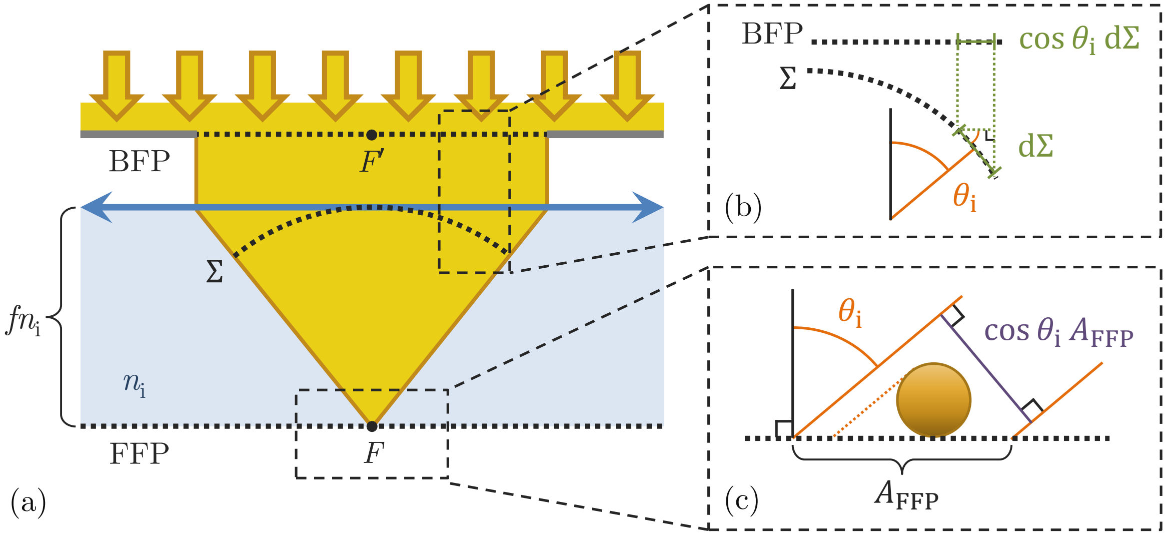

Figure S6. Sectional view of a typical Köhler illumination set-up for micro-spectroscopy experiments. The sketch depicts the main geometrical features associated with an aplanatic condenser lens (double arrow) discussed in the text. The polar angle of incidence $\theta_{\rm i}$ in the magnified insets (b) and (c) is chosen larger that the illumination range in (a) for clarity.

S.IV. INCOHERENT MICROSCOPE ILLUMINATION

In our experiments, incoherent light is focused by a high numerical aperture (NA) condenser lens on the sample, which is thereby illuminated by a range of directions of incidence, as depicted in Fig. S6a. Owing to the symmetry around the optical axis of the microscope, we will refer in the following to this range as to the *illumination cone*. Describing such excitation as a plane wave (PW) impinging perpendicularly onto the sample plane — a common approach to numerical modeling in the nanoplasmonics field — can provide insight on the spectral position and linewidth of the resonant modes of the investigated system, but is not accurate enough to reproduce quantitatively the cross section magnitude and the angular distribution of light scattered to the far-field. For instance, the axial polarisation component introduced by illumination at finite angles of incidence is not accounted for. We therefore develop in this section a mathematical description of high NA, incoherent illumination. In the following sections S.V and S.VI we rely on this description for the cross section measurements in numerical simulations and quantitative data analysis.

A. Analytical description

The scientific-grade condenser lens we use is to a good approximation an aplanatic optical system (henceforth *aplanat*), that is, free from spherical aberration and coma. An aplanat transforms the flat wavefront of a collimated beam in the back focal plane (BFP) into a hemispherical wavefront Σ (often called the *front principal "plane"*) converging at the front

focal point F where the sample is placed, as depicted in Fig. S6a. In our case, however, the illumination originates from an incoherent source and therefore does not form a coherent wavefront in the BFP, nor a point spread function (PSF) in the front focal plane (FFP). In the Köhler illumination scheme we adopt, the coherence length in the BFP is given by the diffraction limit of the illuminated region A_{FFP} in the FFP, which is an image of the field diaphragm. In our experiments, A_{FFP} has a diameter of 500 µm, that is, three orders of magnitude wider than the diffraction-limited PSF size λ/NA for the full condenser NA = 1.34. Accordingly, the coherence length in the BFP is three orders of magnitude shorter than the size of the BFP, which is 28 mm. Now, each of these small coherence patches in the BFP corresponds to a well-defined direction of incidence onto the FFP, identified by the polar angles $(\theta_\text{i}, \varphi_\text{i})$, where the subscript i stands for "incidence" and denotes coordinates and quantities related to the illumination. Therefore, we describe the microscope illumination as an incoherent superposition of PWs having the form derived in Sec. S.II, and impinging onto the sample with directions $(\theta_\text{i}, \varphi_\text{i})$ contained within the illumination cone. In the rest of this section, we derive the dependence on θ_i of the power $\mathrm{d}P_{\text{PW}}$ and intensity $\mathrm{d}I_{\text{PW}}$ carried by each of these PWs.

Let the effective focal length of the condenser (which is the radius of Σ in Fig. S6a) be fn_i, where n_i is the refractive index of the medium filling the FFP space. Note that in our apparatus $fn_\text{i} = 10.5$ mm is twenty times larger than the size of the illuminated region, so that the illumination in the BFP of the condenser is still collimated to a small angular range of approximately 50 mrad. Now, each PW constituting the illumination impinges from a well-defined direction given by the small angular range $\mathrm{d}\Omega_\text{i}$, which identifies an element $\mathrm{d}\Sigma$ of the surface Σ, where the PW can be thought of originating from. The small element of area $\mathrm{d}A_{\text{BFP}}$ in the BFP corresponding to $\mathrm{d}\Sigma$ shrinks towards the edges of the BFP as illustrated by the geometric construction in Fig. S6b

$$\mathrm{d}A_{\text{BFP}} = \cos\theta_\text{i}\,\mathrm{d}\Sigma = (fn_\text{i})^2\cos\theta_\text{i}\,\mathrm{d}\Omega_\text{i} = (fn_\text{i})^2\cos\theta_\text{i}\sin\theta_\text{i}\,\mathrm{d}\theta_\text{i}\,\mathrm{d}\varphi_\text{i}\,. \tag{S25}$$

The $\cos\theta_\text{i}$ factor thereby introduced is characteristic of aplanats and is sometimes referred to as the *aplanatic apodization cosine.*

The result (S25) can also be derived from *Abbe's sine condition*

$$\rho_\text{i} = f\text{NA}_\text{i} = fn_\text{i}\sin\theta_\text{i} \tag{S26}$$

which relates radial position ρ_i in the BFP and polar angle of incidence θ_i in the FFP for an aplanat — and in fact prescribes that the principal plane Σ is a sphere. As for the azimuth φ_i, it is conserved across the condenser since we only consider here axially-symmetric optical elements. Eq. (S25) is obtained by differentiating $\mathrm{d}A_{\text{BFP}} = \rho_\text{i}\,\mathrm{d}\rho_\text{i}\,\mathrm{d}\varphi_\text{i}$ according to Eq. (S26). Incidentally, we observe that the quantity $n^2\cos\theta\sin\theta\,\mathrm{d}\theta$ is conserved across a planar interface as a consequence of Snell's law. This has allowed us to simplify the discussion

by disregarding the interfaces between the condenser immersion medium n_i and the top sample layer n_1.

In a ray picture, the plane wave power $\mathrm{d}P_\mathrm{PW}$ through $\mathrm{d}\Sigma$ is the same power $\mathrm{d}P_\mathrm{BFP}$ crossing $\mathrm{d}A_\mathrm{BFP}$, and thus Eq. (S25) yields

$$\mathrm{d}P_\mathrm{PW} = \mathrm{d}P_\mathrm{BFP} = I_\mathrm{BFP}\,\mathrm{d}A_\mathrm{BFP} = I_\mathrm{BFP}(fn_\mathrm{i})^2\cos\theta_\mathrm{i}\sin\theta_\mathrm{i}\,\mathrm{d}\theta_\mathrm{i}\,\mathrm{d}\varphi_\mathrm{i} \tag{S27}$$

where I_BFP is the illumination intensity over the BFP, which is assumed to be constant. As discussed above, under the Köhler illumination scheme employed in our microscope, the illuminated region A_FFP of the FFP is the image of the field aperture and is the same for all directions of incidence. This implies that the plane wave elements have a wavefront of size A_PW which, according to the geometry illustrated in Fig. S6c, is given by $A_\mathrm{PW} = A_\mathrm{FFP}\cos\theta_\mathrm{i}$, reduced by the *beam squeezing cosine* with respect to A_FFP. This expression of A_PW can be used to calculate the intensity $\mathrm{d}I_\mathrm{PW}$ of the plane wave

$$\mathrm{d}I_\mathrm{PW} = \frac{\mathrm{d}P_\mathrm{PW}}{A_\mathrm{PW}} = \frac{I_\mathrm{BFP}\,\mathrm{d}A_\mathrm{BFP}}{A_\mathrm{FFP}\cos\theta_\mathrm{i}} = \frac{I_\mathrm{BFP}}{A_\mathrm{FFP}}(fn_\mathrm{i})^2\sin\theta_\mathrm{i}\,\mathrm{d}\theta_\mathrm{i}\,\mathrm{d}\varphi_\mathrm{i} \tag{S28}$$

where the expression (S25) of $\mathrm{d}A_\mathrm{BFP}$ has been substituted in the last equality.

Let us summarize the main results obtained in this section. According to Eq. (S27), the plane wave power $\mathrm{d}P_\mathrm{PW} \propto \cos\theta_\mathrm{i}\,\mathrm{d}\Omega_\mathrm{i}$ decreases as θ_i increases, as ruled by the aplanatic apodization cosine. Eq. (S28) shows that the plane wave intensity $\mathrm{d}I_\mathrm{PW} \propto \mathrm{d}\Omega_\mathrm{i}$ is instead independent of the illumination direction: This is a non-trivial consequence of the exact compensation between the aplanatic apodization cosine and the beam squeezing cosine under the Köhler illumination scheme.

B. Set-up-specific corrections

There are a few considerations specific to our set-up which can be included as corrections to the rather general analytical description outlined above. First, the illumination is not completely homogeneous in the BFP as assumed above, due to a non-ideal performance of the diffuser. Specifically $I_\mathrm{BFP}(\rho_\mathrm{i})$ drops towards the edges of the BFP, down to approximately 85 % of the intensity in the center. Second, the transmittance of the condenser drops steeply for $\mathrm{NA}_\mathrm{i} > 1.1$. We ascribe this behavior to stronger reflections from the internal optical interfaces at higher angles of incidence. Both mechanisms — and the second to a larger extent — contribute to diminish $\mathrm{d}I_\mathrm{PW}$ at large θ_i. We have characterized experimentally these effects and discussed them in the supporting information of Ref. [S3]. For the purposes of this work, we limit ourselves to reproduce in Fig. S7 the measured angular dependence of the intensity impinging on the sample, whose trend is explained by the considerations above.

The sample mounting we used for experiments in air features an interface between the glass slide and the top sample layer n_1, see Fig. 1. This interface is not included in the

numerical simulations as it is $\sim 100\lambda$ away from the nano-object; therefore the additional angular dependence of $I_{\rm PW}$ on $\theta_{\rm i}$ due to reflection losses has been included by hand. For unpolarized illumination, the dependence is given by the average of the Fresnel transmittance of the glass/air interface for p and s polarisation: $T(\theta_{\rm i}) = (T_{\rm p} + T_{\rm s})/2$. Qualitatively, the stronger reflection from the interface at large angles dims the illumination at high $\mathrm{NA_i}$.

For notational simplicity, all the corrections affecting the angular dependence $\mathrm{d}I_{\rm PW}(\theta_{\rm i})$ can be lumped into an angular efficiency $\Xi(\theta_{\rm i})$. For the effects considered in this section, its explicit expression would be $\Xi = (\mathrm{d}I_{\rm PW}/\,\mathrm{d}\Omega_{\rm i}) \times T$. The corrected form of Eq. (S28) for our set-up thus reads

$$\mathrm{d}I_{\rm PW} = \frac{I_{\rm BFP}}{A_{\rm FFP}}(fn_{\rm i})^2\,\Xi(\theta_{\rm i})\sin\theta_{\rm i}\,\mathrm{d}\theta_{\rm i}\,\mathrm{d}\varphi_{\rm i}\,. \tag{S29}$$

S.V. QUANTITATIVE CROSS SECTION MODELING

The optical cross sections and the angular distribution of scattered light are computed numerically for each cube with the numerical model described in Sec. S.III. The excitation in these simulations is a PW with a given direction of incidence $(\theta_{\rm i}, \varphi_{\rm i})$, whose analytical expression in a three-layer medium we derived in Sec. S.II. However these numerical results cannot be directly compared to the experimental measurements, since the illumination cone produced by a high NA lens contains a broad range of directions, as already discussed above. Let us therefore distinguish the two cases, and use superscripts to denote the type of excitation, so that $\sigma^{\rm PW}$ and σ^{l} are the cross section under plane wave and microscope illumination respectively. The superscript l indicates a specific illumination cone, determined by the minimum and maximum polar angle of illumination $\underline{\theta}^{l}_{\rm i}$ and $\overline{\theta}^{l}_{\rm i}$. Specifically, our quantitative method is based on correlating a bright-field (BF) and a dark-field (DF) measurement, so that $l \in \{\mathrm{BF, DF}\}$. In this section, we employ the mathematical description

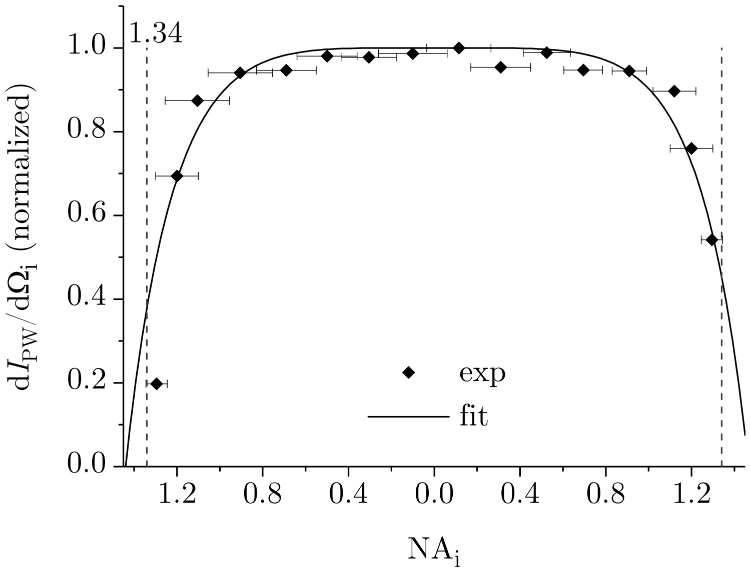

Figure S7. Normalized illumination intensity focused on the sample by the condenser as a function of the numerical aperture of illumination $\mathrm{NA_i}$, measured under the same conditions as the experiments presented in this work. The solid line is the polynomial fit to the data used for analysis. The vertical dashed lines at $\mathrm{NA_i} = 1.34$ indicate the nominal condenser aperture.

of the microscope illumination developed in Sec. S.IV to express σ^l in terms of σ^{PW} and thereby relate simulated and measured cross sections.

Optical cross sections are defined as the power P removed from the exciting electromagnetic radiation by a given optical process divided by the incident intensity I_0: $\sigma \equiv P/I_0$. When the excitation is a single mode — such as a plane wave or a Gaussian beam — the identification of I_0 is straightforward; but in our case of incoherent, high-NA illumination, it is less obvious. We have taken as a reference the intensity I^l_{FFP} incident on the FFP, so that the cross section is defined as $\sigma^l \equiv P^l/I^l_{\text{FFP}}$. This seems the most natural definition in microscopy experiments, where the FFP is imaged onto the sensor and hence the signal measured in transmission is proportional to I^l_{FFP}. Moreover, this definition reduces to the usual plane wave definition in the limit of small illumination NAs.

Now, I^l_{FFP} is the sum of the small intensity contributions $\mathrm{d}I^l_{\text{FFP}} = \cos\theta_{\text{i}}\,\mathrm{d}I_{\text{PW}}$ of all the PWs composing the illumination cone. Note that the intensity projected onto the FFP is lower than the PW intensity I_{PW} by a factor $\cos\theta_{\text{i}}$ according to the geometric construction in Fig. S6c. Similarly, P^l is the sum of the small power contributions $\mathrm{d}P^l = \sigma^{\text{PW}}(\theta_{\text{i}},\varphi_{\text{i}})\,\mathrm{d}I_{\text{PW}}$ removed from all the PWs composing the illumination cone. Putting together these considerations one has

$$\sigma^l \equiv \frac{P^l}{I^l_{\text{FFP}}} = \frac{\int_l \sigma^{\text{PW}}(\theta_{\text{i}},\varphi_{\text{i}})\,\mathrm{d}I_{\text{PW}}}{\int_l \cos\theta_{\text{i}}\,\mathrm{d}I_{\text{PW}}}. \tag{S30}$$

We emphasize that it is correct to add the PW contributions since they are incoherent to each other and thus do not interfere. Finally, since we simulated σ^{PW} as a function of the direction of incidence, it is convenient to recast the integral (S30) over the angular variables using the last form of Eq. (S29)

$$\sigma^l = \frac{\int_{\underline{\theta}^l_{\text{i}}}^{\overline{\theta}^l_{\text{i}}}\int_0^{2\pi}\sigma^{\text{PW}}(\theta_{\text{i}},\varphi_{\text{i}})\,\Xi(\theta_{\text{i}})\sin\theta_{\text{i}}\,\mathrm{d}\theta_{\text{i}}\,\mathrm{d}\varphi_{\text{i}}}{\int_{\underline{\theta}^l_{\text{i}}}^{\overline{\theta}^l_{\text{i}}}\int_0^{2\pi}\cos\theta_{\text{i}}\,\Xi(\theta_{\text{i}})\sin\theta_{\text{i}}\,\mathrm{d}\theta_{\text{i}}\,\mathrm{d}\varphi_{\text{i}}}. \tag{S31}$$

This formula can be used to compute the simulated cross section under microscope illumination, to be directly compared with the experimental spectra. Often the symmetries of the investigated system (excitation + nano-object) can be exploited to reduce the integration domain and hence the number of simulations required without loss of information. Specifically, the cubes under unpolarized illumination investigated in this work display an eight-fold symmetry and it is therefore sufficient to cover the azimuthal domain $\varphi_{\text{i}} \in [0,\pi/4]$. The directions of incidence we averaged with Eq. (S31) are determined by a square grid in the BFP of the condenser, which is identified by the NA coordinates $(\text{NA}_x, \text{NA}_y) = (n_{\text{i}}\sin\theta_{\text{i}}\cos\varphi_{\text{i}},\ n_{\text{i}}\sin\theta_{\text{i}}\sin\varphi_{\text{i}})$. The grid has 13 points along the coordinate axes of the BFP (which has a radius of $n_{\text{i}} - 0.01$), corresponding to steps in NA of 0.2517 for measurements in anisole ($n_{\text{i}} = 1.52$) and 0.1650 for measurements in air ($n_{\text{i}} = 1.00$).

For averaging directions of incidence equidistant in NA_x, NA_y (rather than in $\theta_{\text{i}}, \varphi_{\text{i}}$) the

integration must be recast in terms of $\mathrm{d}A_{\text{BFP}} = f^2\,\mathrm{dNA}_x\,\mathrm{dNA}_y$ by substituting into Eq. (S30) the second to last expression of (S28)

$$\sigma^l = \frac{\int_{A^l_{\text{BFP}}} [\sigma^{\text{PW}}(\theta_\text{i}, \varphi_\text{i}) / \cos\theta_\text{i}]\, \mathrm{d}A_{\text{BFP}}}{\int_{A^l_{\text{BFP}}} \mathrm{d}A_{\text{BFP}}} = \left\langle \frac{\sigma^{\text{PW}}}{\cos\theta_\text{i}} \right\rangle_{A^l_{\text{BFP}}} \tag{S32}$$

which, for simplicity, does not include the set-up specific corrections introduced in Sec. S.IV B (*i. e.* $\Xi = 1$). Notably, Eq. (S32) offers itself to a rather straightforward interpretation: σ^l is the average of σ^{PW} over the illuminated region A^l_{BFP} of the BFP, weighted by $\cos\theta_\text{i}$ to account for the projection of the incident intensity onto the FFP. Effectively, the way referencing is performed in experiments enhances the contribution of high NAs and thereby brings about $\sigma^l > \sigma^{\text{PW}}$, as if the shadow projected by the nano-object onto the FFP was measured (dotted orange line in Fig. S6c). By virtue of this analogy, we call such dependence of the measured cross section on the illumination the *long shadow effect*. We have verified experimentally this effect in Ref. [S4], and observed a good agreement with our theoretical predictions.

S.VI. QUANTITATIVE CROSS SECTION MEASUREMENT

Our group has recently reported in Ref. [S3] an experimental method for measuring quantitatively — that is, in absolute units — the cross sections of optical scattering and absorption of a single nano-object (henceforth object). In this method, the optical signals detected experimentally are quantified into cross sections relying on the knowledge of four parameters, see Eq. (7) in Ref. [S3]. In this section we describe how these parameters are calculated for the cubes investigated in this work. Note that all parameters are wavelength dependent and calculated accordingly, but this is omitted to unburden the notation.

A. Scattering parameters η^l and ζ

$\eta^l \equiv P^l_{\text{obj}}/P^l_{\text{tot}}$ is defined as the ratio of the scattered power collected by the objective to the total scattered power. $\zeta \equiv P^{\text{BF}}_{\text{tot}}/P^{\text{DF}}_{\text{tot}}$ is defined as the BF-to-DF ratio of the total scattered power. The power scattered within a certain directional range can be written as an integral of the angular distribution $\mathcal{P}^l_{\text{FF}}$ of power scattered by the object to the far-field

$$P^l_{\text{sca}}(\underline{\theta}^l_\text{d}, \overline{\theta}^l_\text{d}) = \int_{\underline{\theta}^l_\text{d}}^{\overline{\theta}^l_\text{d}} \int_0^{2\pi} \mathcal{P}^l_{\text{FF}}(\theta, \varphi)\, \sin\theta\, \mathrm{d}\theta\, \mathrm{d}\varphi \tag{S33}$$

where the conical range of detection is delimited by the polar angles $\left[\underline{\theta}^l_\text{d}, \overline{\theta}^l_\text{d}\right]$, while we assumed full axial collection $\varphi \in [0, 2\pi)$ by the objective. Note that we are reserving the calligraphic glyph $\mathcal{P}$ to angular power densities. Let $\theta_{\text{obj}} = \pi - \arcsin(\text{NA}_{\text{obj}}/n_3)$ be the polar angle defining the acceptance of the objective of $\text{NA} = \text{NA}_{\text{obj}}$ in medium 3 (where the

integration occurs). The *scattering parameters* defined above can be expressed in terms of the integral (S33) as

$$\eta^l = \frac{P^l_{\text{sca}}(\theta_{\text{obj}},\pi)}{P^l_{\text{sca}}(0,\pi)} \quad \text{and} \quad \zeta = \frac{P^{\text{BF}}_{\text{sca}}(0,\pi)}{P^{\text{DF}}_{\text{sca}}(0,\pi)}\,. \tag{S34}$$

In Ref. [S3] $\mathcal{P}^l_{\text{FF}}$ is computed analytically by describing the object as a collection of dipoles with orientations determined by the illumination cone. This approach simplifies the automation of the analysis, and thus the usage of the method. On the other hand, several approximations are involved in the dipole representation. Instead, in this work, $\mathcal{P}^l_{\text{FF}}$ is computed using the far-field transform built in COMSOL so to achieve the widest applicability domain and best accuracy. In particular, this approach is also appropriate for larger objects (*i. e.* of size $\gtrsim \lambda/10$) where the electrostatic approximation would not be accurate, and does not resort to dipole tensorial forms of the polarisability of the object. We have already calculated in Sec. S.II the analytical form of the exciting field $\boldsymbol{E}_{\text{exc}}$ to be used in simulations with PW illumination. Following the blueprint laid down in Sec. S.IV, we reproduce the incoherent microscope illumination by averaging the results of many PW simulations with directions of incidence spanning the experimental illumination cone. Let us now show in detail how this scheme applies to the calculation of the scattering parameters.

In each direction in space identified by the polar angles (θ,φ), the radiated power density $\mathcal{P}^l_{\text{FF}}$ is the sum of all PW contributions $\mathcal{P}^{\text{PW}}_{\text{FF}}$ within the illumination cone l

$$\mathcal{P}^l_{\text{FF}} = \int_l \mathcal{P}^{\text{PW}}_{\text{FF}} \frac{\mathrm{d}I_{\text{PW}}}{I_0} \tag{S35}$$

where I_0 is the intensity of the exciting PW in the simulations. The integral can be rewritten in terms of the illumination angular variables θ_{i}, φ_{i} via Eq. (S29)

$$\mathcal{P}^l_{\text{FF}}(\underline{\theta}^l_{\text{i}},\overline{\theta}^l_{\text{i}}) \propto \int_{\underline{\theta}^l_{\text{i}}}^{\overline{\theta}^l_{\text{i}}} \int_0^{2\pi} \mathcal{P}^{\text{PW}}_{\text{FF}}(\theta_{\text{i}},\varphi_{\text{i}})\,\Xi(\theta_{\text{i}})\sin\theta_{\text{i}}\,\mathrm{d}\theta_{\text{i}}\,\mathrm{d}\varphi_{\text{i}} \tag{S36}$$

where we have omitted all constants since the scattering parameters (S34) are computed as ratios of the integral (S33). As already discussed with reference to Eq. (S31), the azimuthal integration domain can be reduced by exploiting the symmetry of the investigated system. Eventually, the scattering parameters are computed by substituting Eq. (S36) into Eq. (S33), and then into Eq. (S34).

B. Illumination parameters ξ and τ

Quantitative cross section measurements require as well two *illumination parameters*, which depend exclusively on the illumination configuration of the experiment, but not on the optical properties of the measured nano-object.

The first parameter, called ξ, is defined as the BF-to-DF ratio of the reference power I^l_{FFP}.

This can be written in the same way as the denominator of Eq. (S30)–(S31) so that

$$\xi \equiv \frac{I_{\mathrm{FFP}}^{\mathrm{BF}}}{I_{\mathrm{FFP}}^{\mathrm{DF}}} = \frac{\int_{\underline{\theta}_{\mathrm{i}}^{\mathrm{BF}}}^{\overline{\theta}_{\mathrm{i}}^{\mathrm{BF}}} \cos\theta_{\mathrm{i}}\, \Xi(\theta_{\mathrm{i}}) \sin\theta_{\mathrm{i}}\, \mathrm{d}\theta_{\mathrm{i}}}{\int_{\underline{\theta}_{\mathrm{i}}^{\mathrm{DF}}}^{\overline{\theta}_{\mathrm{i}}^{\mathrm{DF}}} \cos\theta_{\mathrm{i}}\, \Xi(\theta_{\mathrm{i}}) \sin\theta_{\mathrm{i}}\, \mathrm{d}\theta_{\mathrm{i}}} \tag{S37}$$

where we have exploited the axial symmetry of the illumination to remove the integration on the azimuthal coordinate φ_{i}. Note that if the set-up-specific corrections introduced in Sec. S.IV B are not taken into account, Eq. (S37) reduces to a simple analytical expression

$$\xi\Big|_{\Xi=1} = \frac{\sin^2\overline{\theta}_{\mathrm{i}}^{\mathrm{BF}} - \sin^2\underline{\theta}_{\mathrm{i}}^{\mathrm{BF}}}{\sin^2\overline{\theta}_{\mathrm{i}}^{\mathrm{DF}} - \sin^2\underline{\theta}_{\mathrm{i}}^{\mathrm{DF}}} = \frac{A_{\mathrm{BFP}}^{\mathrm{BF}}}{A_{\mathrm{BFP}}^{\mathrm{DF}}}. \tag{S38}$$

In the last equality we highlighted that ξ is the ratio of the illuminated areas in the BFP: This occurs because without corrections I_{BFP} is homogeneous over the condenser aperture in the BFP, and the condenser has a flat angular response.

As discussed in Sec. S.V, under microscope illumination the cross sections are referenced to the intensity I_{FFP}^{l} incident on the FFP in medium 1. The transmission signal of an empty region of the sample measured in a BF configuration is proportional to $(I_{\mathrm{FFP}}^{\mathrm{BF}})_{3}^{\mathrm{FF}}$, which is the intensity traversing the FFP transmitted to medium 3 and propagating to the far field (FF). These two intensities are related via the second illumination parameter defined as $\tau^{\mathrm{BF}} \equiv (I_{\mathrm{FFP}}^{\mathrm{BF}})_{3\mathrm{f}}^{\mathrm{FF}}/(I_{\mathrm{FFP}}^{\mathrm{BF}})_{\mathrm{i}}$, whereby the transmittance of the sample grid is accounted for. Now, for each PW of the illumination cone, the ratio of transmitted to incident intensity is

$$|t_{13}|^2 \equiv \frac{(\mathrm{d}I_{\mathrm{PW}})_{3\mathrm{f}}}{(\mathrm{d}I_{\mathrm{PW}})_{\mathrm{i}}} = \left[\frac{(\mathrm{d}I_{\mathrm{PW}})_{3\mathrm{fp}} + (\mathrm{d}I_{\mathrm{PW}})_{3\mathrm{fs}}}{(\mathrm{d}I_{\mathrm{PW}})_{\mathrm{ip}} + (\mathrm{d}I_{\mathrm{PW}})_{\mathrm{is}}}\right] = \frac{n_3}{n_1}\left[\frac{|E_{3\mathrm{fp}}|^2 + |E_{3\mathrm{fs}}|^2}{|E_{\mathrm{ip}}|^2 + |E_{\mathrm{is}}|^2}\right] \tag{S39}$$

with the field amplitudes computed in Sec. S.II. In the second equality of Eq. (S39), the p and s polarized contributions have been averaged to reproduce unpolarized illumination. Eventually, the analytical expression of τ^{BF} is the sum over the BF illumination cone of all incoherent PW intensity contributions projected onto the FFP

$$\tau^{\mathrm{BF}} = \frac{\int_{\mathrm{BF}} \mathrm{Re}(\cos\theta_{3\mathrm{f}})(\mathrm{d}I_{\mathrm{PW}})_{3\mathrm{f}}}{\int_{\mathrm{BF}} \cos\theta_{\mathrm{i}}\, \mathrm{d}I_{\mathrm{PW}}} = \frac{\int_{\underline{\theta}_{\mathrm{i}}^{\mathrm{BF}}}^{\overline{\theta}_{\mathrm{i}}^{\mathrm{BF}}} |t_{13}(\theta_{\mathrm{i}})|^2\, \mathrm{Re}(\cos\theta_{3\mathrm{f}})\, \Xi(\theta_{\mathrm{i}}) \sin\theta_{\mathrm{i}}\, \mathrm{d}\theta_{\mathrm{i}}}{\int_{\underline{\theta}_{\mathrm{i}}^{\mathrm{BF}}}^{\overline{\theta}_{\mathrm{i}}^{\mathrm{BF}}} \cos\theta_{\mathrm{i}}\, \Xi(\theta_{\mathrm{i}}) \sin\theta_{\mathrm{i}}\, \mathrm{d}\theta_{\mathrm{i}}} \tag{S40}$$

where we substituted with Eq. (S39) and Eq. (S29) in the last equality. The real part of $\cos\theta_{3\mathrm{f}}$ is taken to implement the FF projection, as the cosine is imaginary for evanescent PWs.

S.VII. SPECTROSCOPY OF ALL INVESTIGATED CUBES

Fig. 3 of the paper displays the quantitatively measured and simulated optical cross section spectra of a single exemplary cube (# 4). The same quantities are plotted in the figures S8–S10 for all the 11 cubes we investigated. The individual TEM micrograph of each cube is displayed as an inset. In a few cases in anisole, the value ζ/ξ in the analysis was adjusted slightly to reduce negative absorption values, to compensate apparent variations in the illumination. The spectra are all rather similar between different cubes, indicating a good precision (*i. e.* reproducibility) of the quantitative microspectroscopy. The peak position λ_{D} and the full width at half maximum Δ_{D} of the D resonance in anisole are given in Table S1, for both experiment and simulation.

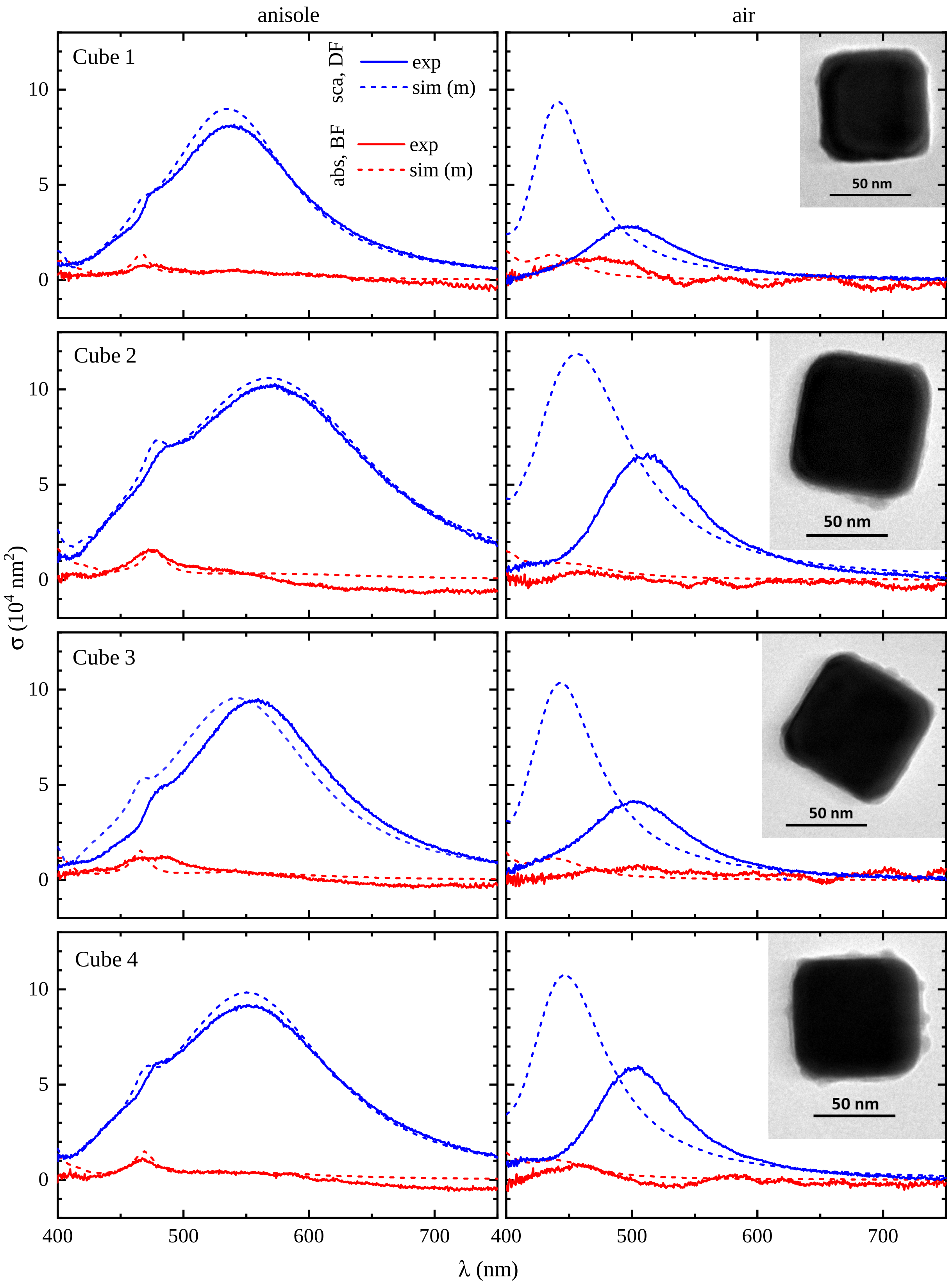

Figure S8. Measured and simulated optical cross section spectra of the investigated cubes 1 to 4, immersed in anisole (left) or in air (right). The corresponding TEM images are shown as insets.

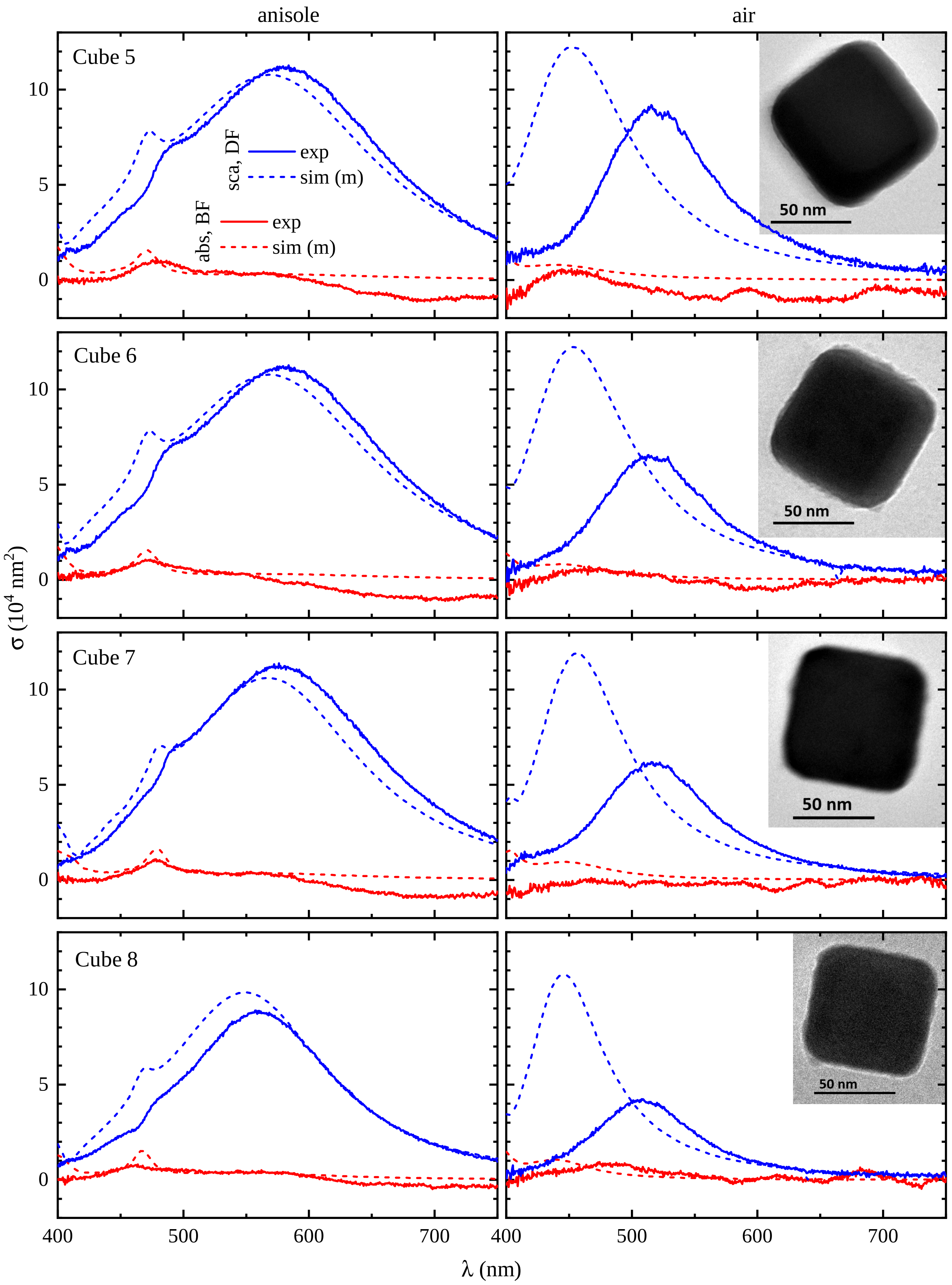

Figure S9. Same as Fig. S8, but for cubes 5 to 8.

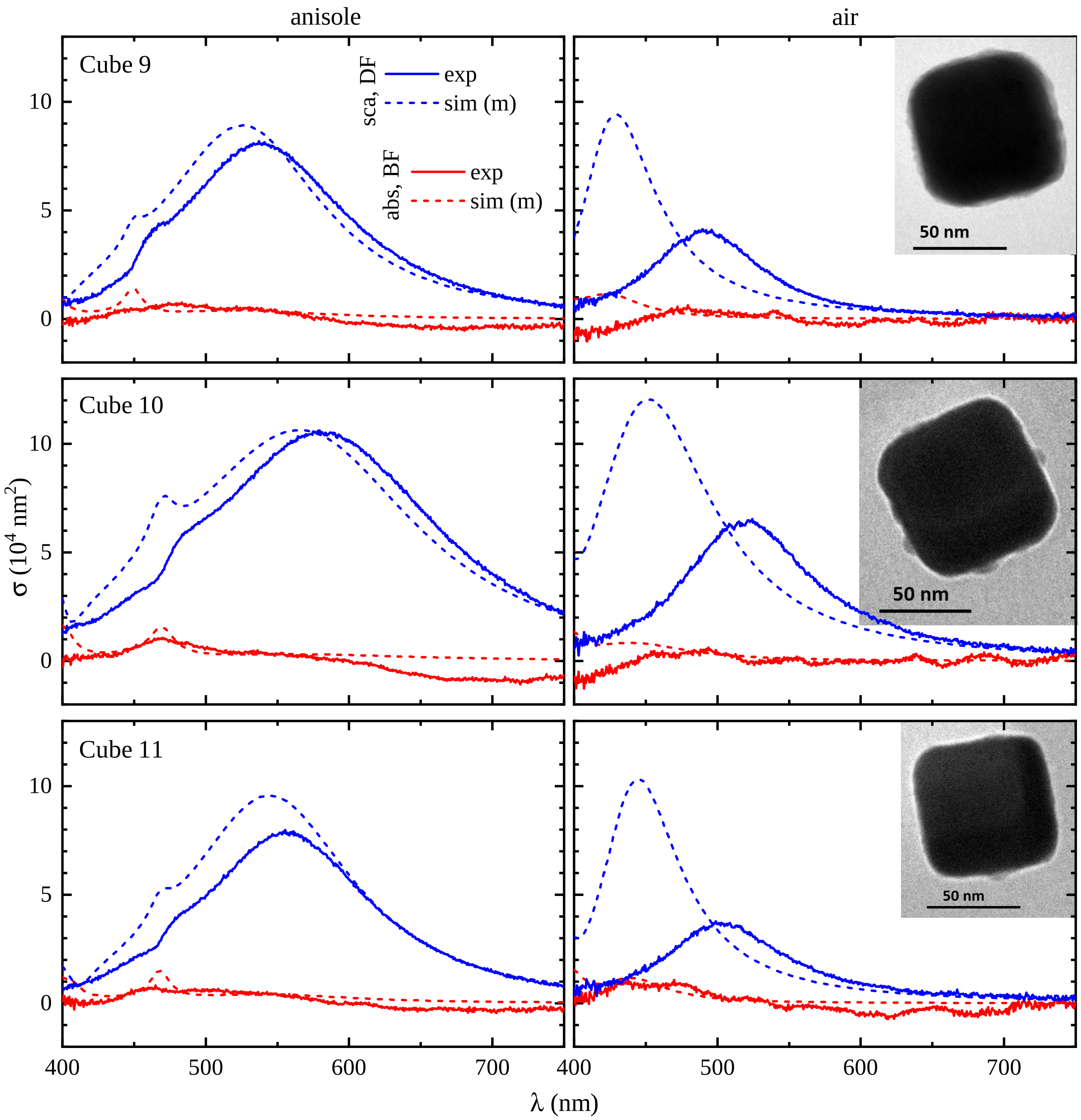

Figure S10. Same as Fig. S8, but for cubes 9 to 11.

S.VIII. CORRELATION OF CUBE-ATTACHED MATERIAL WITH SCATTERING SPECTRA

The TEM images of the cubes in Fig. S8, Fig. S9, and Fig. S10 show some material adhered irregularly to the cube surface, which could be the tarnish discussed in the main text. We note that a previous study of photooxidation of Ag NPs showed TEM images with similar features in the early stages (see Fig. 5a in Ref. [S5]), while sulfidation of Ag cubes did not produce such irregular features (see Fig. 3a in Ref. [S6]).

Here, we correlate the amount of adhered material, which we hypothesise to be tarnish, with the scattering cross-section spectra measured in air. To quantify the amount of material, we threshold the TEM images as exemplified in Fig. S11a for cube 1. We extract the area A_c of the Ag core using a threshold at 40% of the range between the modes of the brightness histogram of core and background, while for the area A_t of the Ag core and tarnish, we use a threshold of 80% . From these areas, we extract the core size as $\sqrt{A_c}$, and the tarnish thickness accordingly as $(\sqrt{A_t} - \sqrt{A_c})/2$. In order to remove the systematic dependencies on the core size, we describe these by λ_{fit} for the peak wavelength λ_{LSPR} as shown in Fig. S11b, and by σ_{fit} for the corresponding cross-section σ_{sca}^{DF}, as shown in Fig. S11c. Notably, the tarnish thicknesses extracted are in the range of 1.2-2.5 nm, consistent with the thicknesses required to explain the spectra as discussed in Fig. 6. While λ_{LSPR} does not show a correlation with the tarnish thickness, see Fig. S11d, when removing the core size systematics in the variation $\lambda_{LSPR} - \lambda_{fit}$, given in Fig. S11e, some correlation is found, however opposite to the expected trend. σ_{sca}^{DF} does not show a correlation with the tarnish thickness, see Fig. S11f, and also the normalized cross-section $\sigma_{sca}^{DF}/\sigma_{fit}$, given in Fig. S11g, does not show correlation.

We note that the observed material distribution is irregular, and far from a surface layer of homogeneous thickness. It is therefore expected that the effective tarnish layer thickness we extract from the TEM images is not accurately representing the effect of the material, assuming it is tarnish, on the measured cross-section spectra. Specifically, the Q and D modes have different field distributions across the cube surface, and are thus sensitive to the detailed three-dimensional arrangement of the tarnish. The rather weak correlations observed in Fig. S11e and Fig. S11g are therefore expected, even if the hypothesis that the observed material is tarnish is correct.

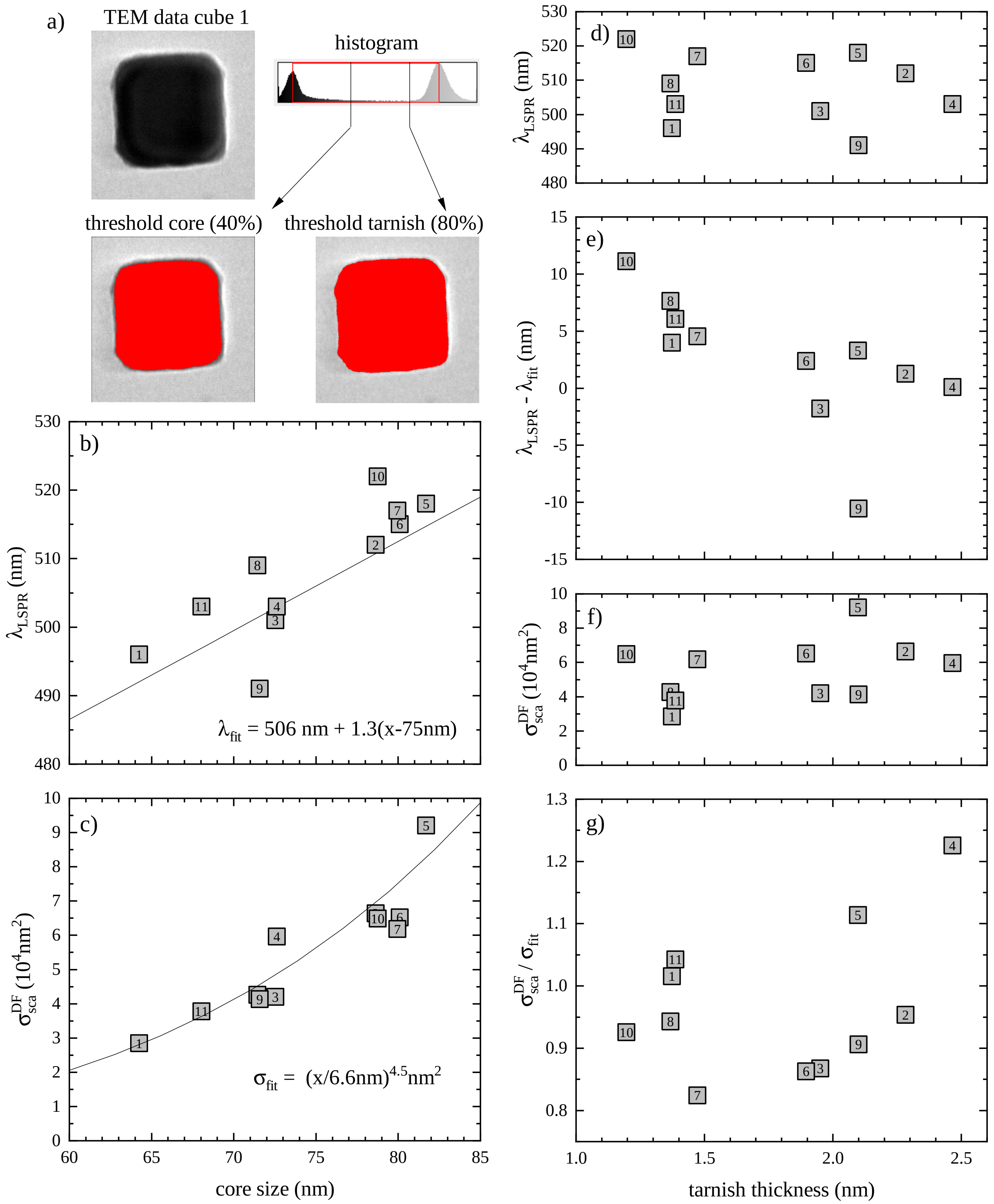

Figure S11. Analysis of tarnish seen in TEM and its correlation with the scattering spectra. a) Example of procedure to quantify the effective thickness of the tarnish: the TEM image (top) is thresholded to select the area A_c of the Ag core only (left), and the area A_t of the Ag core and the surrounding tarnish (right). b) Peak wavelength λ_{LSPR} of σ_{sca}^{DF} as function of core size determined as $\sqrt{A_c}$, with a fit λ_{fit} as shown. c) Peak cross-section σ_{sca}^{DF} as function of core size, with a fit σ_{fit} as shown. d) λ_{LSPR} as function of tarnish thickness determined as $(\sqrt{A_t} - \sqrt{A_c})/2$. e) Variation $\lambda_{LSPR} - \lambda_{fit}$ as function of tarnish thickness. f) σ_{sca}^{DF} as function of tarnish thickness. g) Normalized cross-section $\sigma_{sca}^{DF}/\sigma_{fit}$ as function of tarnish thickness.

[S1] Y. Wang, *Fabrication and Quantitative Correlative Light-Electron Microscopy of Novel Plasmonic Nanoparticles*, Ph.D. thesis, Cardiff University, School of Biosciences (2019).

[S2] M. E. Marques, A. A. Mansur, and H. S. Mansur, Appl. Surf. Sci. **275**, 347 (2013).

[S3] A. Zilli, W. Langbein, and P. Borri, ACS Photonics **6**, 2149 (2019).

[S4] L. Payne, A. Zilli, Y. Wang, W. Langbein, and P. Borri, Proc. SPIE **10892**, Colloidal Nanoparticles for Biomedical Applications XIV, 108920J (2019).

[S5] N. Grillet, D. Manchon, E. Cottancin, F. Bertorelle, C. Bonnet, M. Broyer, J. Lermé, and M. Pellarin, J. Phys. Chem. C **117**, 2274 (2013).

[S6] C. Fang, Y. H. Lee, L. Shao, R. Jiang, J. Wang, and Q.-H. Xu, ACS Nano **7**, 9354 (2013).
